# Structural design of microbicidal cationic oligomers and their synergistic interaction with azoles against *Candida albicans*

**DOI:** 10.1038/s41598-019-48322-x

**Published:** 2019-08-15

**Authors:** Yuan Yuan, Feng Zhou, Haibin Su, Yugen Zhang

**Affiliations:** 1 Institute of Bioengineering and Nanotechnology, 31 Biopolis Way, The Nanos, Singapore, 138669 Singapore; 2 School of Materials Science and Engineering, Nanyang Technological University, 50 Nanyang Avenue, Singapore, 639798 Singapore; 3 Department of Chemistry, The Hong Kong University of Science and Technology, Hong Kong, China

**Keywords:** Medicinal chemistry, Structure-based drug design

## Abstract

Membrane-disrupting synthetic antimicrobial polymers have been well developed as antimicrobial peptide (AMP) mimics to mitigate antimicrobial resistance (AMR). However, synthetic polymers possess inherent drawbacks, being a mixture of different chain lengths, which restricts their clinical applications. In fact, synthetic oligomers with defined chain length and molecular structure could be better representatives of AMPs. Herein, a series of novel imidazolium-ammonium oligomers developed in this work exhibit excellent broad spectrum antimicrobial activity, specifically the salient structure dependent high efficiency against *C*. *albicans*. Moreover, synergistic effect emerged when the combined azoles and synthetic oligomers were applied against *C*. *albicans*. The detail structural coupling between azoles and oligomers was scrutinized through molecular dynamics simulations to unravel the interaction details with the atomistic resolution. The labile interaction between oligomer and azoles facilitated the transfer of drug into fungal cells, which can be a synergistic solution to prevent the development of resistance on *C*. *albicans*.

## Introduction

Antimicrobial resistance (AMR) is one of the major global healthcare threats^1,2^. Its development is closely related to the overuse of antibiotics, including clinical and non-therapeutic applications^3^. The emergence of resistance against antibiotics is hardly surprising as antibiotics generally inhibit specific intracellular processes and resistance can be acquired by mutations in targets or related processes^4^. Antimicrobial peptides (AMPs) kill microbe via electrostatic and hydrophobic interactions which disrupt the lipid domains of cytoplasmic membrane^5^. This membrane-disrupting mechanism could prevent the development of resistance^6^. However, the high cost of manufacture, proteolytic degradation and *in vivo* toxicity limit the clinical application of AMPs. To overcome these problems, synthetic antimicrobial polymers have been well studied as AMP mimics^7–10^. Synthetic polymers were designed to have similar amphiphilic structures non-specific membrane-targeting activity to AMPs^11–16^. And a few AMP mimics displayed potent antifungal activity^17–21^. Despite the excellent *in vitro* and *in vivo* antimicrobial activities, there is concern for the clinical application of synthetic polymers about the heterogeneity (a mixture of polymers with different chain lengths) and toxicity related to high molecular weight component. Instead, synthetic oligomers with defined chain length and molecular structure could be a better representation of AMPs^22–27^. The structure parameters of oligomers, such as balanced amphiphilicity, charge density and structural flexibility, could be tailored to achieve optimum antimicrobial functionality and biocompatibility. Herein, a series of novel imidazolium-ammonium oligomers was developed. These oligomers exhibited excellent broad spectrum antimicrobial activities, including a structure dependent high activity against *C*. *albicans*.

Candidiasis is one of the most prevalent human opportunistic fungal infections among immunocompromised or debilitated patients^28,29^. The largest family of antifungal drugs are azoles. Azoles are only fungi-static and their efficacy relies on the presence of cellular host defenses^30^. Moreover, with the prolonged and extensive clinical use of azoles, drug-resistance emerged rapidly^31,32^. These problems underlie the need for novel antifungal agents or improved therapeutic strategies. Considering that the development of new antifungal drugs is relatively slow, more attentions have been paid to combination therapy^33^. The antifungal combinations could enhance the rate and extent of killing, and widen the spectrum and potency of drug activity, achieving a more rapid antifungal effect and also reducing the dosage of individual drugs. In some cases, they could also minimize the development of antifungal resistance or reduce toxicity. Currently, fluconazole, the most widely used azole drug, has been studied in combination with antibiotics^34–42^ and a few non-antifungal compounds^43–45^ against *C*. *albicans*. In this study, combinations of azoles and synthetic oligomers were tested on *C*. *albicans* and the structure dependent synergistic effects were established and further investigated with molecular dynamics simulations.

## Results and Discussion

### Structural design of cationic oligomers

**Figure 1:**
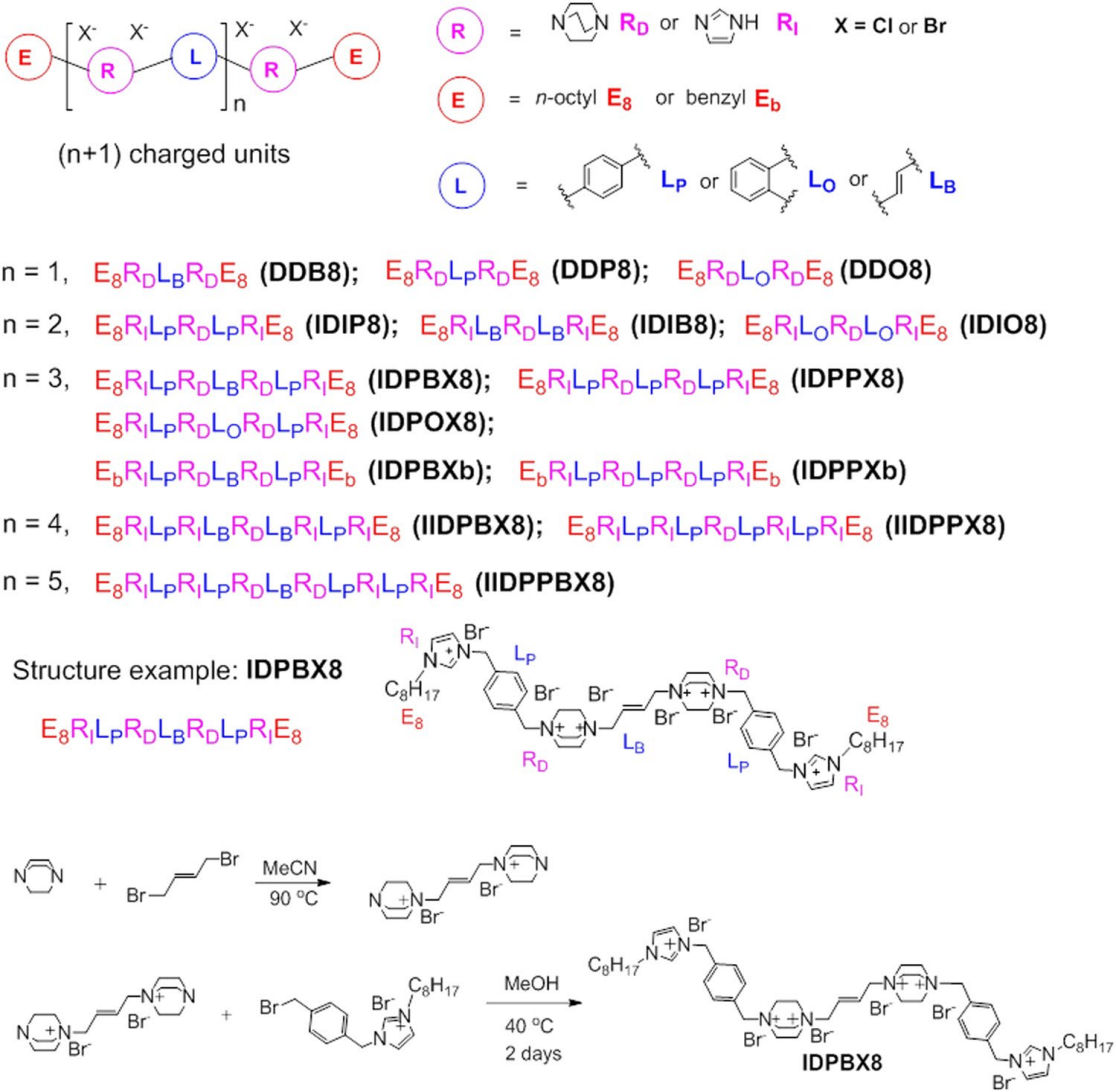
General formula, charge units, structure codes and structures of ammonium-imidazolium oligomers. Structure and synthesis scheme of IDPBX8.

Both imidazole^46–49^ and 1,4-diazabicyclo-[2.2.2]-octane (DABCO)^50–53^ have been used as basic units to synthesize antimicrobial materials. Imidazole is a planar aromatic molecule. When both of the nitrogen atoms are substituted, the resulting imidazolium will bear a single positive charge. DABCO is a steric flexible molecule which will bear two positive charges when the two nitrogen atoms are both substituted. Herein, imidazole and DABCO are selected as synthons to construct oligomers with suitable linkers and ending groups to achieve optimum antimicrobial property by tuning their charge density, hydrophobicity and structure flexibility (Fig. 1).

### Structure-activity relationship

The antimicrobial activities of oligomers were evaluated against four different clinically relevant microbes: *S*. *aureus*, *E*. *coli*, *P*. *aeruginosa*, and *C*. *albicans*, as presented in Table 1. In general, the properties of these ammonium-imidazolium oligomers are correlated to their structural components, such as linkers, ending groups, and the chain length/the number of charged units. The structure of the ending group is crucial for the antimicrobial activity. All the oligomers except **IDPPXb** exhibited strong antibacterial activity against *S*. *aureus* and *E*. *coli*. **IDPPXb**, which possesses benzyl as the ending group, showed very weak activity against the four microbes. The result was in accordance with literature that the long aliphatic ending group can enhance antimicrobial activity by facilitating the interaction between the antimicrobial compound and the cell membrane^26^.

**Table 1:** Antimicrobial activity (MIC, µg mL^−1^), the fractional inhibitory concentration index (FIC) with Fluconazole, haemolytic property (HC_10_, µg mL^−1^), and critical micelle concentration (CMC, µg mL^−1^) of the ammonium-imidazolium oligomers.

Sample name	MIC (µg mL^−1^)^a^				HC_10_^b^ (µg mL^−1^)	FIC^c^	CMC (µg mL^−1^)
	*S*.*A*.	*E*.*C*.	*P*.*A*.	*C*.*A*.			
DDB8	4	4	125	125 (16)	>2000	0.25	1552
DDP8	8	4	2000	500 (31)	>2000	0.19	1896
DDO8	4	2	31	125 (16)	>2000	0.38	3038
IDIP8	2	4	500	125 (8)	>2000	0.25	985
IDIB8	8	16	500	>500 (31)	>2000	0.25	1829
IDIO8	4	4	500	62 (31)	>2000	0.25	1667
IDPBX8	2	2	62	8–31 (2–8)	>2000	0.38	1456
IDPPX8	4	8	125	62 (8–31)	>2000	0.25	1552
IDPOX8	4	8	62	125 (16)	>2000	0.25	1367
IDPBXb	31	16	500	>1000 (1000)	>2000	na	1732
IDPPXb	62	62	1000	2000 (500)	na	0.5	1860
ITPPX8	4	4	500	125 (16)	>2000	0.25	1342
IIDPBX8	4	62	250	62 (8)	>2000	0.31	291
IIDPPX8	1	2	62	125 (16)	>2000	0.38	451
IIDPPBX8	2	4	62	31 (2–8)	>2000	1.00	727
IBN-C8^17^	4	8	16	16 (2–4)	>2000	2.00	226
Fluconazole	—	—	—	>125 (2–4)	—	—	—
Itraconazole	—	—	—	>2 (0.016–0.032)	—	—	—
Voriconazole	—	—	—	>2 (0.025–0.10)	—	—	—

*EC* (*E*. *coli*), *SA* (*S*. *aureus*), *P A* (*P*. *aeruginosa*), *CA* (*C*. *albicans*), Flu (fluconazole), Itra (itraconazole), Vori (voriconazole).

^a^MIC tested against ~10^6^ CFU mL^−1^ of microbes (MIC tested at ~10^8^ CFU mL^−1^ of microbes was included in SI, Table S1). The MIC was taken as the lowest concentration of the antimicrobial oligomer that no visible growth was observed by unaided eyes. For MIC tested against *C*. *albicans*, the lowest concentration that inhibited at least 50% fungal growth was listed in brackets.

^b^HC_10_ was taken as oligomer concentration at which the oligomer causes 10% hemolysis.

^c^The FIC of a compound with fluconazole was calculated using the lowest concentration of a compound that inhibited at least 50% fungal growth since fluconazole is fungal-static.

Besides the ending group, the central linker is also very important for their performance. When the number of charged units is 4, **IDPOX8** which has *o*-xylenyl linker in the center exhibited the greatest antibacterial activity compared to those with *p*-xylenyl linker or *trans*-butene linker. However, **IDPBX8**, with a more flexible butene linker, was the most effective antifungal compound with MIC of 8–31 µg mL^−1^ against *C*. *albicans*. A similar trend was observed when the number of charged units is 2. **DDO8** with *o*-xylenyl linker displayed lowest MIC against the three bacteria.

In addition, the length of the main-chain oligomers or the number of charged units also affects their antimicrobial property. The chain lengths of **DDB8**, **IDPBX8** and **IIDPPBX8** are different although they have similar structure components. The shortest **DDB8**, with the highest charge density, showed greatest antimicrobial activity against *S*. *aureus* and *E*. *coli*, while the long chain molecules with flexible butene linker, **IDPBX8** and **IIDPPBX8**, were more active toward *C*. *albicans*.

The oligomers could easily form cationic micelles in water (see Table 1). Considering that their critical micelle concentrations (CMCs) are in the range of 291 to 3038 μg mL^−1^ which is much higher than their effective antimicrobial concentrations, the micelle formation may not have significant influence on their microbicide property. In addition, the haemolytic behaviour of the oligomers was tested over a range of concentrations (Table 1). All the oligomers did not induce noticeable haemolysis even at the highest concentration 2000 μg mL^−1^. Considered alongside their high antimicrobial activity, these oligomers are primarily qualified as active and non-toxic compounds which display high selectivity for a wide range of pathogenic microbes over mammalian cells.

**Figure 2:**
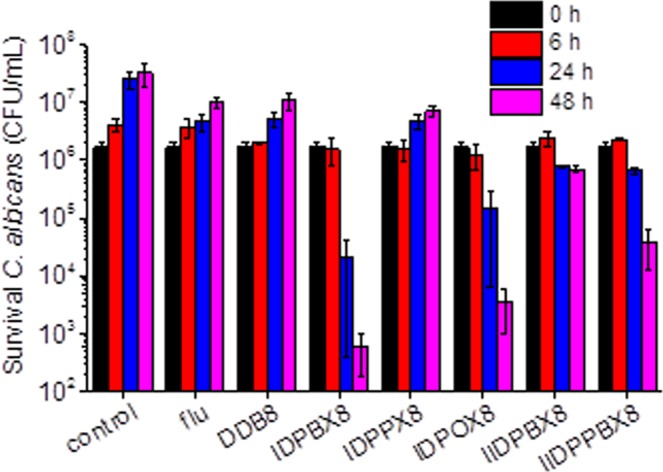
Antifungal activity of selected oligomers and fluconazole (flu) against *C*. *albicans*. Colony forming unit of *C*. *albicans* after treatment with oligomers at 62 μg mL^−1^ for different periods. *C*. *albicans* grown in Yeast Mold broth were used as control. The data are expressed as mean ± S.D. of triplicates.

The killing efficacy of selected oligomers was evaluated against *C*. *albicans* at 62 μg mL^−1^ with fluconazole as positive control (Fig. 2). After 24 h treatment with fluconazole, the growth of *C*. *albicans* was suppressed, indicating the fungi-static nature of fluconazole. All of the synthetic oligomers also exhibited antifungal activity. Specifically, **IDPPX8** and **DDB8** were found to be fungi-static while the other oligomers were fungicidal. **IDPBX8** killed *C*. *albicans* faster than others. 99% killing was observed after 24 h treatment and it increased to 99.99% after 48 h.

### Synergistic effect with azoles against *C. albicans*

**Figure 3:**
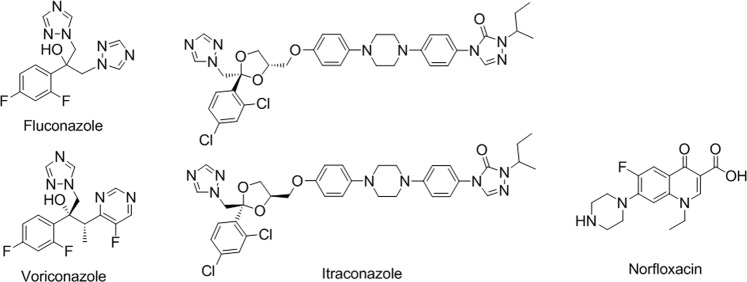
Structure of azoles and norfloxacin.

Azoles, more specifically triazoles, are currently the most widely used and studied class of antifungal agents. To achieve enhanced efficacy, the interaction of our synthetic oligomers with norfloxacin (an antibiotic drug for bacterial infection) and three triazoles, including fluconazole, intraconazole and voriconazoles (Fig. 3) were investigated.

**Figure 4:**
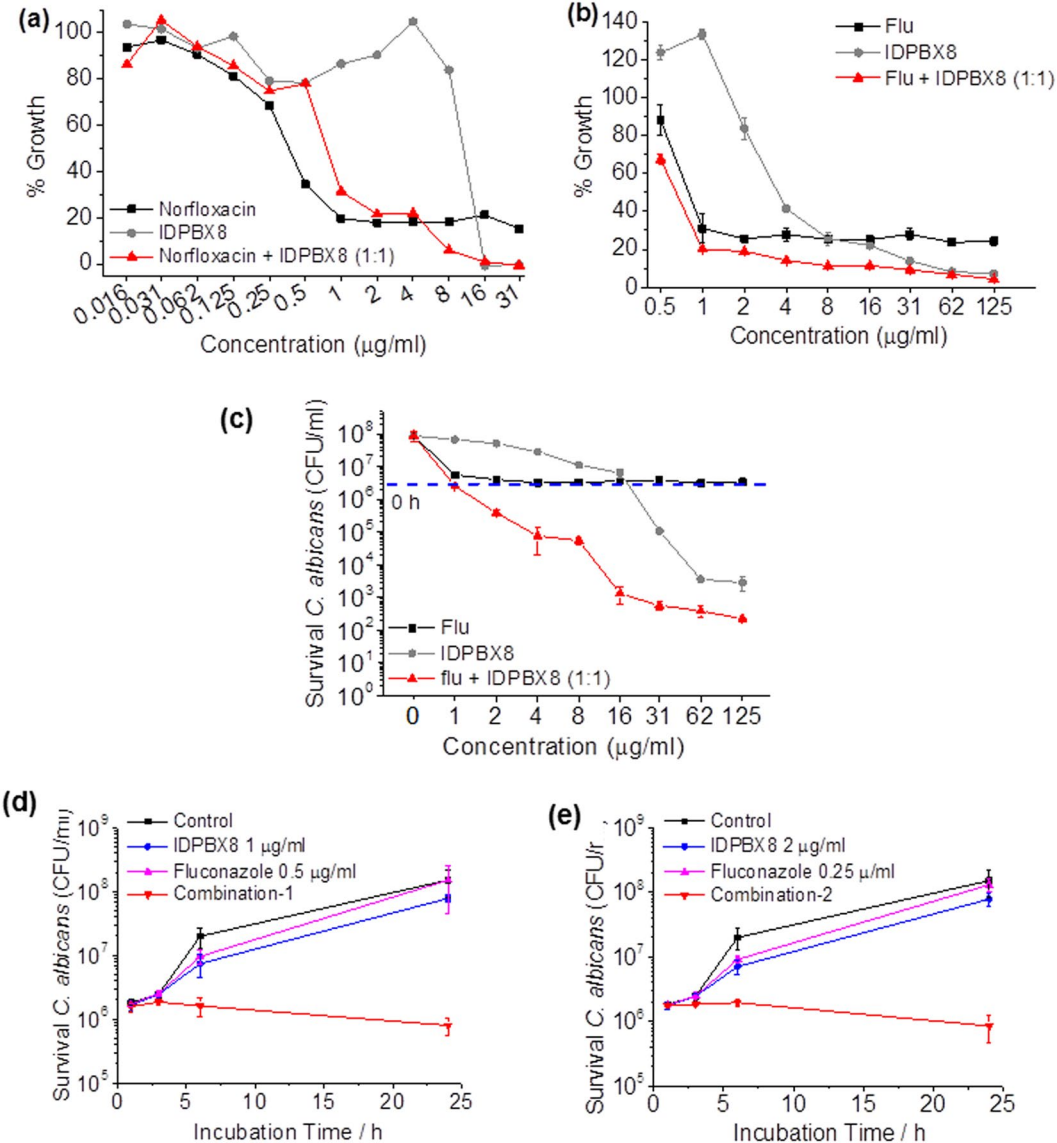
Synergistic effect was observed between **IDPBX8** and fluconazole, but not norfloxacin. (**a**) Growth of *S*. *aureus* after 24 h incubation with varied concentration of norfloxacin, **IDPBX8** and their mixture. (**b**) Growth of *C*. *albicans* after 24 h incubation with varied concentration of fluconazole, **IDPBX8** and their mixture. The % growth was calculated based on the absorbance at 600 nm measured by a plate reader. (**c**) Colony forming units (CFU) of *C*. *albicans* after 24 h incubation with varied concentration of fluconazole, **IDPBX8** and their mixture. The concentration of *C*. *albicans* at 0 h is 3.8 × 10^6^ CFU mL^−1^ as indicated in the figure as blue dash line. (**d**,**e**) Time-kill curve of **IDPBX8**, fluconazole alone and in combination against *C*. *albicans*, colony forming units (CFU) of *C*. *albicans* at different time after incubation with **IDPBX8** or fluconazole alone, or combinations. (**d**) Combination-1: **IDPBX8** (1 μg mL^−1^) and fluconazole (0.5 μg mL^−1^); (**e**) Combination-2: **IDPBX8** (2 μg mL^−1^) and fluconazole (0.25 μg mL^−1^). The data are expressed as mean ± S.D. of triplicates.

Firstly, the interactions of **IDPBX8** with norfloxacin or fluconazole were studied. The growth of microbes was monitored by measuring turbidity and quantified with colony counting method. From Fig. 4a,b, the combination of **IDPBX8** and norfloxacin (1:1 wt ratio) did not show improved efficacy against *S*. *aureus* while **IDPBX8** combined with fluconazole showed higher activity against *C*. *albicans* than each compound/medicine used alone. The results of colony counting using nutrient agar plates reflected the concentration of survival *C*. *albicans* (Fig. 4c). Killing effect was observed when the total concentration of the combination is as low as 2 μg mL^−1^, while killing was only observed when the concentration of individual component (the oligomer used alone) reached 31 μg mL^−1^.

The interaction of the oligomers with triazoles was further studied using checkerboard dilution and time kill methods^54^. The fractional inhibitory concentration (FIC) index values for the interaction of oligomers with fluconazole were reflected in Table 1. Oligomers with less than 6 charged units (n < 5) and *n*-octyl ending group all gave synergistic profiles when they were combined with fluconazole at sub-inhibitory concentrations. And synergistic effects were observed with at least one dose pair of combination against *C*. *albicans*. On the contrary, oligomers with 6 charged units (n = 5) and oligomers with aromatic ending groups all resulted in indifferent effects (0.5 < FIC ≤ 4). No antagonistic effects were found in all of the combinations.

Time-kill experiments were performed with selected antifungal combinations according to the results of the checkerboard assay. Figures 4d,e illustrate the synergistic kinetics of combinations of **IDPBX8** and fluconazole in the time kill assay. Combination of 1 μg mL^−1^ **IDPBX8** plus 0.5 μg mL^−1^ fluconazole, and of 2 μg mL^−1^ **IDPBX8** plus 0.25 μg mL^−1^ fluconazole revealed a stable and continuous inhibition of colony counts after 24 h compared to the single substance of **IDPBX8** or fluconazole.

The interaction of oligomers with other triazoles, such as itraconazole and voriconazole, was also investigated using checkerboard dilution method. The FICs of different combinations against *C*. *albicans* were shown in Table 2. Synergy was also observed when **IDPBX8** or **IDPPX8** was applied together with voriconazole.

**Table 2:** Effect of treatments with combinations of selected oligomers and triazoles on the growth of *C*. *albicans* according to the FIC.

Components		MIC^b^ (µg mL^−1^)		Combined MIC (µg mL^−1^)		FIC
A	B^a^	A	B	A	B	
IDPBX8	Flu	8	2	1	0.5	0.38
IDPBX8	Itra	8	0.03	1	0.015	0.62
IDPBX8	Vori	8	0.1	2	0.006	0.31
IDPPX8	Flu	16	4	2	0.25	0.19
IDPPX8	Itra	16	0.016	4	0.004	0.50
IDPPX8	Vori	16	0.025	4	0.003	0.30
IIDPPBX8	Flu	2	4	1	2	1.0

^a^Flu (fluconazole), Itra (itraconazole), Vori (voriconazole).

^b^MICs were the lowest concentration that inhibited at least 50% fungal growth.

Three types of mechanism are frequently cited as resulting in synergy between two or more antifungal agents^33^. The first mechanism results from sequential inhibition of different steps of a common biochemical pathway. The second proposed mechanism is simultaneous inhibition of cell wall and cell membrane targets in fungi. The third mechanism arises from simultaneous therapy with cell wall or cell membrane-active agents to enhance the penetration of a second antifungal agent. Herein, it was observed that synergistic effect occurred between fluconazole and short chain oligomers with charge units less than 6. When the charge unit of oligomer chain is 6 or more, synergy was not observed although individual components had antifungal property. These results cannot be simply explained by the three reported mechanisms. It is plausible that the interactions between azoles and oligomers are essential to their synergistic interaction.

To further understand the mechanism of these synergistic effects, interactions between oligomers and drug molecules were studied by molecular dynamics simulations^55,56^. Oligomers, **DDP8** (n = 1), **IDIP8** (n = 2), **IDPBX8**, **IDPPX8**, **IIDPBX8** (n = 4), **IIDPPBX8** and **IBN-C8** (n = 5) were selected for the molecular dynamics study.

**Figure 5:**
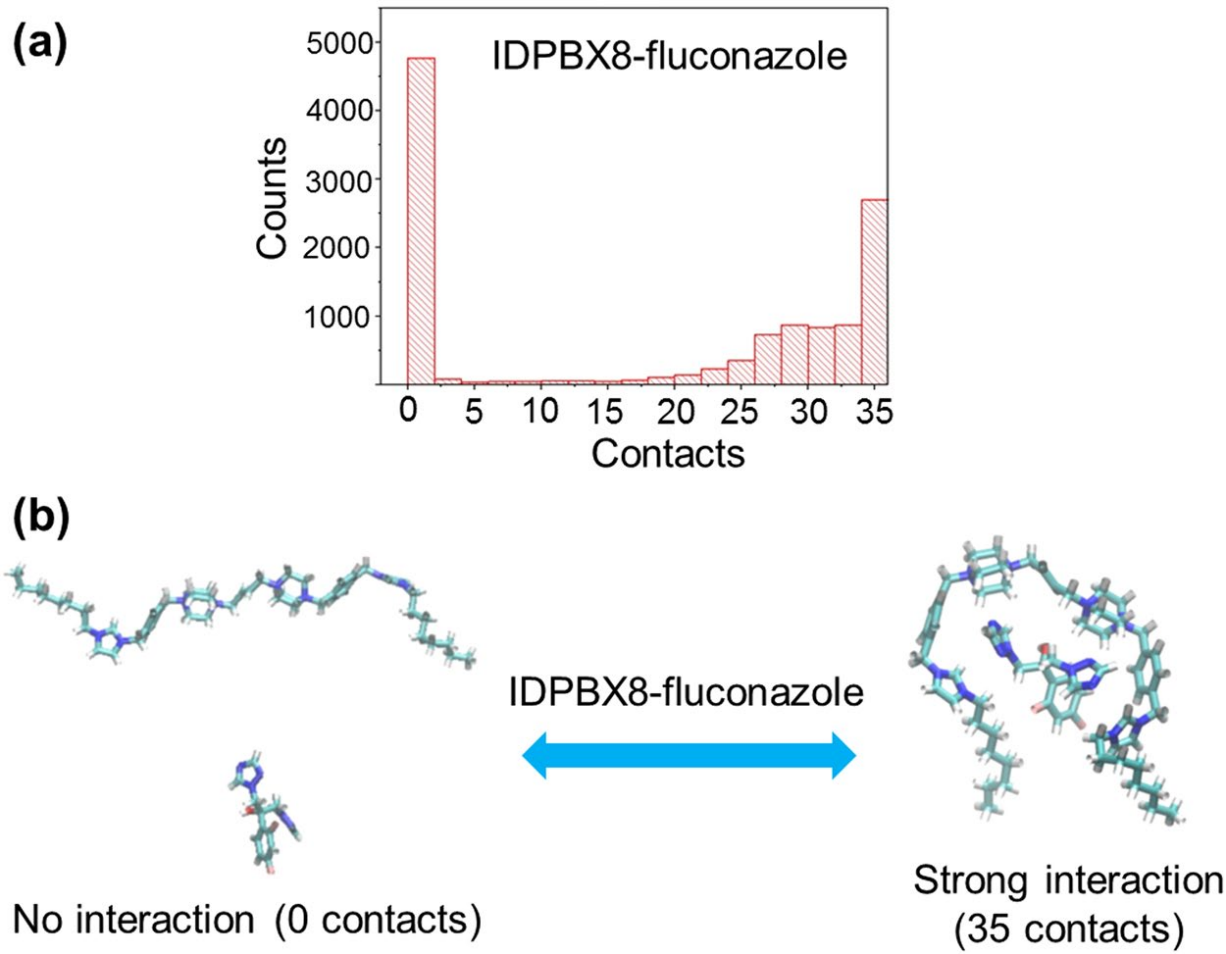
Interactions between **IDPBX8** (A) and fluconazole (B) calculated by molecular dynamics modeling. (**a**) Contacts is the number of atom-atom contact within 0.6 nm between A and B. Contacts at 0 means A and B has no atom-atom contacts within 0.6 nm, in other words, A and B are separated. Counts at 35 means A and B has 35 atom-atom contacts within 0.6 nm, implying A and B are attached. (**b**) The figure shows molecular structures of “separated” and “attached” status and their dynamic equilibrium.

The interactions between oligomers with charge units less than 6 (n < 5) and the fluconazole molecules are very labile. Figures 5 and S1–2 illustrates the equilibrium between contacted oligomer-fluconazole and separated dual. Oligomers with 6 charge units (n = 5) have much stronger interaction with fluconazole molecules. They are mostly tightly coupled in the course of simulation of 20 ns long (Table 3). The labile interaction between oligomer and drug molecule promotes synergistic effect between them. These interactions are anticipated to allow oligomers to attack cell membrane, and subsequently, facilitate the fluconazole molecules to pass through the cell membrane. In contrast, the synergistic effect disappeared for those oligomer/fluconazole combinations with very strong interactions. Interestingly, antagonistic effect was also absent for these strong interactive combinations. The strong interaction between oligomer and drug molecule may hinder the release of the drug into the inner cell through translocation across the cell membrane. Similar phenomena were also observed for oligomers with other drug molecules. **IDPBX8** had labile interactions with voriconazole, but strong interactions with itraconazole. Experimentally, synergistic effect was observed for **IDPBX8** and voriconazole combination, while no synergy was observed for **IDPBX8** and itraconazole.

**Table 3:** The fractional inhibitory concentration index (FIC) and computational interactions between oligomers and azoles.

Sample name	Charge units	Azoles	Interaction Index^a^	FIC
DDP8	2	Fluconazole	0.85	0.19
IDIP8	3	Fluconazole	0.09	0.25
IDPBX8	4	Fluconazole	0.44	0.38
IDPPX8	4	Fluconazole	0.61	0.25
IIDPBX8	5	Fluconazole	0.27	0.31
IIDPPBX8	6	Fluconazole	0.005	1.00
IBN-C8	6	Fluconazole	0	2.00
IDPBX8	4	Voriconazole	0.65	0.31
IDPBX8	4	Itraconazole	0	0.62

^a^Contact points during 20 ns trajectory between oligomers and azole molecules were counted in molecular dynamics simulations. Interaction index: counts less than 20 contacts/total counts. Interaction index less than 0.01 means very strong interactions between oligomers and azole molecules.

### Drug resistance study

**Figure 6:**
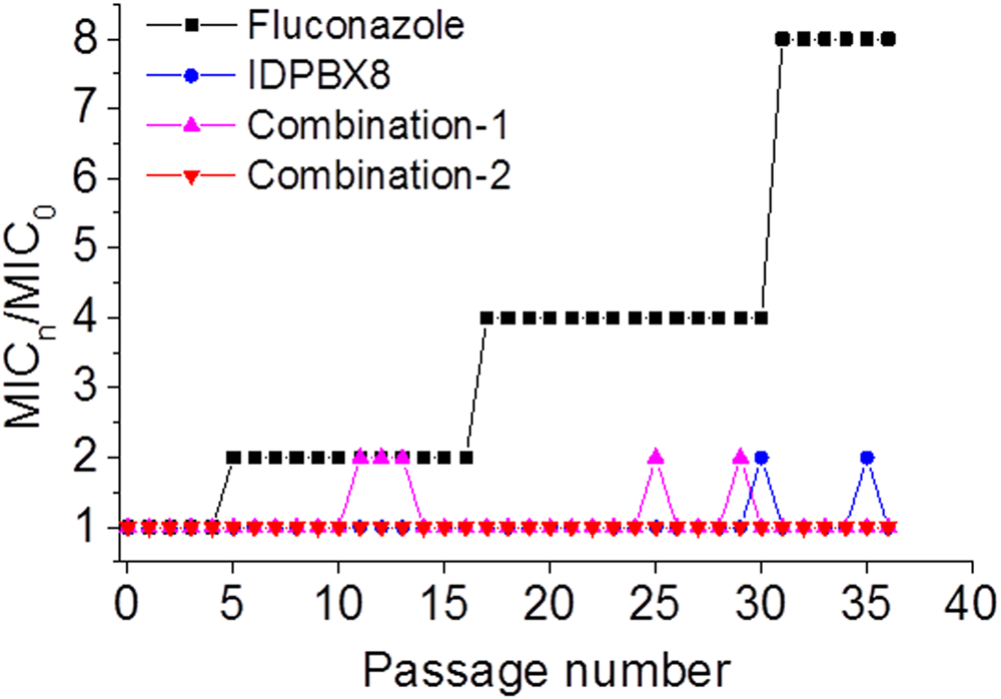
Resistance acquisition in the presence of ¼MIC levels of **IDPBX8**, fluconazole or fluconazole-**IDPBX8** combinations against *C*. *albicans*. Combination-1: **IDPBX8** (1 μg mL^−1^) and fluconazole (0.5 μg mL^−1^); Combination-2: **IDPBX8** (2 μg mL^−1^) and fluconazole (0.25 μg mL^−1^).

Resistance to antifungal agents has been much less studied compared to antibacterial resistance^57^. However, the current increase in fungal infections has intensified the exploration of innovative, safer and more efficient agents to combat fungal infections. The potential for *C*. *albicans* to develop resistance following repeated exposures to **IDPBX8** or fluconazole-**IDPBX8** combinations was investigated by serial passage of *C*. *albicans* treated under ¼MIC levels for oligomer or combinations. MIC values were measured after each passage. For comparison, fluconazole was also tested. As shown in Fig. 6, the MIC of fluconazole against *C*. *albicans* increases at the 5^th^ passage. By the 31^th^ passage the MIC of fluconazole increases to 8 times of the original MIC. In contrast, the MICs of **IDPBX8** and fluconazole-**IDPBX8** combinations are relatively stable over the entire 36 passages, indicating that no significant resistance was developed by *C*. *albicans* during the 36 consecutive days of treatment with **IDPBX8** or fluconazole-**IDPBX8** combinations.

As we observed in Fig. 4, repeated use of oligomers alone would not induce resistance as their working mechanism involves directly destroying cell wall/membrane. Similarly, the combinations of oligomer and fluconazole could also prevent the development of resistance as the main target of their synergistic therapy is the cell membrane too. In other words, the synergistic combination therapy accelerates the delivery of fluconazole into the cell without changing its action targets.

In summary, a series of imidazolium-ammonium oligomers were synthesized and their antimicrobial activities were evaluated against bacteria and fungus. These oligomers generally have broad spectrum antimicrobial property. Among them, oligomers with more flexible linkers demonstrated higher efficiency against *C*. *albicans*. Structure-dependent synergistic interaction of oligomers with azoles was also established. The labile interaction between oligomer and azoles is desirable in the transfer of drug into fugal cells, thus promoting the synergistic effect. The membrane-disruption mechanism ensures oligomers and oligomers/azoles combinations to be protected from resistance development. The results reported here demonstrate a new series of promising imidazolium-ammonium oligomers which can be applied in combination with fluconazole for the treatment of fungal infections in a safer and more effective way. Our study also provides new insight into the mechanism of drug synergy interaction.

## Methods

### Minimum inhibitory concentration

*Staphylococcus aureus* (ATCC 6538, Gram-positive), *Escherichia coli* (ATCC 8739, Gram-negative), *Pseudomonas aeruginosa* (ATCC 9027, Gram-negative), and *Candida albicans* (ATCC 10231, fungus) were used as representative microorganisms to challenge the antimicrobial functions of the oligomers. All bacteria and fungus were frozen at −80 °C, and were grown overnight at 37 °C in Mueller Hinton Broth (MHB, BD Singapore) prior to experiments. Fungus was grown overnight at 22 °C in Yeast Mold Broth (YMB, BD Singapore)^27,58^. Subsamples of these cultures were grown for a further 3 h and diluted to give an optical density (O.D.) value of 0.07 at 600 nm (OD600 = 0.07), corresponding to 3 × 10^8^ CFU mL^−1^ for bacteria and 10^6^ CFU mL^−1^ for fungus (McFarland’s Standard 1; confirmed by plate counts).

The oligomers were dissolved in MHB at a concentration of 4 mg mL^−1^ and the minimal inhibitory concentrations (MICs) were determined by microdilution assay. Bacterial solutions (100 μL, 10^6^ CFU mL^−1^) were mixed with 100 μL of oligomer solutions (normally ranging from 4 mg mL^−1^ to 2 µg mL^−1^ in serial two-fold dilutions) in each well of the 96-well plate. The plates were incubated at 37 °C for 24 h with constant shaking speed at 300 rpm. The MIC measurement against *Candida albicans* was similar to bacteria except that the fungus solution was in YMB and the plates were incubated at room temperature.

The MIC was taken as the lowest concentration of the antimicrobial oligomer that no visible growth was observed by unaided eyes. For MIC test against *C*. *albicans*, the lowest concentration that causes at least 50% inhibition in cell growth comparing to untreated control (prominent decrease in turbidity, or score of 2, according to CLSI guidelines^59^) was also recorded for further synergy study. Cell growth (i.e., the OD_600_) was monitored with a microplate reader (TECAN Spark® 10 M, Austria). Medium solution containing microbial cells alone was used as a control (100% microbial growth). The assay was performed in four replicates and the experiments were repeated at least two times.

### Monitoring the growth of *C. albicans*

The growth of *C*. *albicans* in the presence of **IDPBX8** and fluconazole alone or their combination was monitored by measuring the absorbance at 600 nm with a microplate reader and quantified using colony counting method. Briefly, material was dissolved in YMB (2 µg mL^−1^ to 62 µg mL^−1^ in serial two-fold dilution). A hundred microliters of each solution were placed into a 96-well plate. Then 100 µL of *C*. *albicans* suspension (7.6 × 10^6^ CFU mL^−1^) was added into each well. So the final concentration of *C*. *albicans* was 3.8 × 10^6^ CFU mL^−1^. Fungus growing in pure YMB was used as control. The 96-well plate was kept on a shaker at room temperature under constant shaking. After incubation for 24 h, the absorbance of the solutions (OD600) was measured with a plate reader and used for the calculation of the % growth using the absorbance of control solution as 100% growth. To quantify the number of viable fungi, after 24 h incubation, aliquots (100 µg mL^−1^) were withdrawn and serially diluted with DPBS buffer (1:10). 100 μL of each dilution were spread onto nutrient agar plate (Luria-Bertani broth with 1.5% agar) and the colony forming units (CFU) were counted after incubation at room temperature for 2 days.

### Checkerboard assay

In order to evaluate whether individual oligomer compounds exhibit synergy or indifference in combination with fluconazole against *C*. *albicans*, checkerboard assays were performed as described previously with slight modification^60^. Two-fold serial dilution of oligomers and fluconazole was prepared in YMB at 4 times the strength of the final concentration ranging from 1/16 to two times of the MIC. Aliquots of 50 μL of each component at a concentration of 4 times the targeted final concentration were mixed in a 96-well plate. A row and a column in which a serial dilution of each agent was present alone were also prepared for MIC test. Then each solution in the well plate was inoculated with 100 μL of logarithmically grown 10^6^ cells mL^−1^ *C*. *albicans* cells in the 96-well plate. The plate also contained a column with *C*. *albicans* only as control (100% cell growth). The cells were incubated at room temperature for 24 h with constant shaking, after which cell growth (i.e., the OD600) was monitored with a plate reader.

Synergy between fluconazole and oligomers was determined by calculating the fractional inhibitory concentration index (FIC). Since fluconazole is fungal-static, the MIC used for FIC calculation was the lowest concentration of an oligomer or fluconazole that caused at least **50**% reduction of cell growth. FIC was calculated as follows: FIC = (MIC_oligomer A in combination_/MIC_oligomer A alone_) + (MIC_azole B in combination_/MIC_azole B alone_). FIC values of ≤0.5, >0.5 to ≤4.0 and >4.0 indicated synergy, indifference, or antagonistic interactions for different combinations.

### Determination of critical micelle concentration

Pyrene solution in acetone (10 µL, 6.16 × 10^−5^ M) was added into glass vials, following which acetone was evaporated at room temperature^61^. Oligomer solution in DI water (1 mL) with various concentrations ranging from 0.01 to 1000 µg mL^−1^ was added into the glass vials, giving a final pyrene concentration of 6.16 × 10^−7^ M and the solutions were kept overnight. The excitation spectra of the solutions were scanned from 300 nm to 360 nm with an emission wavelength of 395 nm. Both the excitation and emission bandwidths were set at 2.5 nm. The intensity ratios (I_337_/I_334_) were plotted against oligomer concentration. The CMC value was given by the intersection of the tangent to the curve at the inflection and the tangent of the points at low oligomer concentrations.

### Time-kill method

Time-kill experiments were performed with selected antifungal combinations based on the results of the checkerboard assay^47,48^. **IDPBX8** and fluconazole were tested alone and in combination at sub-MIC level (bellow original MIC values). The mixtures were inoculated with *C*. *albicans* and adjusted to give a final concentration of about 10^6^ CFU mL^−1^. After 1, 3, 6 and 24 h incubation at room temperature, the respective cell suspensions were collected (100 μL), serially diluted as 1:10, and 100 μL of each dilution was spread on Luria-Bertani agar. Colonies were counted after 48 h incubation at room temperature and the CFU mL^−1^was calculated accordingly.

### Resistance studies

Drug resistance was induced by treating *C*. *albicans* repeatedly with **IDPBX8**, **IDPBX8**-fluconazole combination or fluconazole^58^. Firstly, MICs of the tested compounds were determined against *C*. *albicans* using the broth microdilution method. Then, serial passaging was initiated by transferring microbial suspension grown at the sub-MIC of the copolymers (1/4 of MIC at that passage) for another MIC assay. After 24 h incubation, cells grown at the 1/4-MIC of the test compounds were once again transferred and assayed for MIC. The MIC was tested for 36 passages. Drug-resistant behavior was evaluated by recording the changes in the MIC normalized to that of the first passage.

### Haemolysis study

Fresh mouse red blood cells (RBCs) were diluted with PBS buffer to give a RBC stock suspension (4 vol% blood cells)^58^. 100 μL aliquots of RBC suspension were mixed with 100 µL oligomer solutions of various concentrations (ranging from 4 mg mL^−1^ to 2 µg mL^−1^ in serial two-fold dilutions in PBS). After 1 h incubation at 37 °C, the mixture was centrifuged at 2000 rpm for 5 min. Aliquots (100 µL) of the supernatant were transferred to a 96-well plate. Haemolytic activity was determined as a function of haemoglobin release by measuring absorbance of the supernatant at 576 nm using a microplate reader. A control solution that contained only PBS was used as a reference for 0% haemolysis. Absorbance of red blood cells lysed with 0.5% Triton-X was taken as 100% haemolysis. The data were expressed as mean and S.D. of four replicates.

% Haemolysis = [OD_576nm (polymer)_ − OD_576nm (PBS)_]/[OD_576nm (Triton-X)_ − OD_576nm (PBS)_] × 100%

HC_10_ was taken as oligomer concentration at which the oligomer causes 10% hemolysis.

### Statistical analysis

Data were expressed as means ± standard deviation of the mean (S.D. is indicated by error bars)^58^. Student’s *t*-test was used to determine significance among groups. A difference with p < 0.05 was considered statistically significant.

### Computational method

All molecular dynamics simulations were performed using the GROMACS 4.5.3 suite of programs^27,47^. The amber03 force field was used to describe the drug and oligomer molecules. Water molecules were described using the TIP5P water model. The overall temperature of 300 K was modulated with Nose-Hoover thermostat. The integration of the equations of motion was performed by using a leap frog algorithm with a time step of 1 fs. Periodic boundary conditions were implemented in all systems. A cutoff of 1 nm was implemented for the Lennard–Jones and the direct space part of the Ewald sum for Coulombic interactions. The Fourier space part of the Ewald splitting was computed by using the particle-mesh-Ewald method, with a grid length of 0.16 nm on the side and a cubic spline interpolation. Each sample was run for 20 ns. The initial coordinates of the drug and oligomer molecules were made from Gaussview and optimized by density functional theory using M06-2X functional and 6–31 g(d) basis set as built in Gaussian 09 package.

## Supplementary information

Supporting Information
