# Supplementary material for: Structural design of microbicidal cationic oligomers and their synergistic interaction with azoles against Candida albicans
Source: Sci Rep. 2019 Aug 15;9:11885. doi: 10.1038/s41598-019-48322-x (PMC6695401; doi:10.1038/s41598-019-48322-x)
Supplement: Supplementary file 1 — Supporting Information [file 41598_2019_48322_MOESM1_ESM.doc]

**Supporting Information**

**Structural design of microbicidal cationic oligomers and their synergistic interaction with azoles against *Candida albicans***

*Yuan Yuan,^#1^ Feng Zhou,^#2^ Haibin Su,*^3^ Yugen Zhang*^1^*

**MATERIALS AND METHODS**

**General Information:** All solvents were purchased from Sigma-Aldrich and used without further purification. All other reagents were used as received, except where otherwise noted in the experimental text. ^1^H and ^13^C nuclear magnetic resonance (NMR) spectra were recorded on Bruker AV-400 (400 MHz) spectrometer. Chemical shifts (δ) are reported in parts per million (ppm) with the residual solvent peak of tetramethylsilane used as the internal standard at 0.00 ppm. ^1^H NMR data are reported in the following order: chemical shift, multiplicity (br = broad, s = singlet, d = doublet, t = triplet, q = quartet and m = multiplet), integration and assignment.

**Synthesis of the Oligomers**

**Scheme S1.** Synthesis procedure of **DDB8, DDP8** and **DDO8**.

**Synthesis of a:** A solution of bromooctane (1.0 eq, 0.77 g in THF) was added dropwise to a solution of 1,4-diazabicyclo[2.2.2]octane (DABCO, 5.0 eq, 2.24 g in THF) at 60 °C. After stirring at 60 °C for 24 hours, THF was removed under vacuum and the resulting white solids were washed with diethyl ether 2 times. Then the solids were purified by re-precipitation using methanol/ether. **a** was obtained quantitatively as colorless liquid (>95% yield).

**a: ^1^H NMR** (400 MHz, DMSO-d_6_): δ 3.28 (t, 6H), 3.18 (t, 2H), 3.02 (t, 6H), 1.65 (m, 2H), 1.28 (m, 10H), 0.87 (t, 3H).

**Synthesis of DDB8, DDP8 and DDO8:** A solution of **a** (2.5 eq, 0.47 g in DMF) was mixed with a solution of trans-1,4,-dibromobut-2-ene (1.0 eq, 0.12 g in DMF). After stirring at 60 °C for 48 hours, DMF was removed under vacuum and the resulting white solids were washed with acetone 2 times and diethyl ether 2 times. **DDB8** was obtained quantitatively as white solid (>95% yield). The synthesis of **DDP8** and **DDO8** was similar to **DDB8**.

**DDB8: ^1^H NMR** (400 MHz, DMSO-d_6_): δ 6.40 (s, 2H), 4.41 (d, 4H), 3.99 (m, 24H), 3.55 (t, 4H), 1.72 (m, 4H), 1.31 (m, 20H), 0.89 (t, 6H).

**
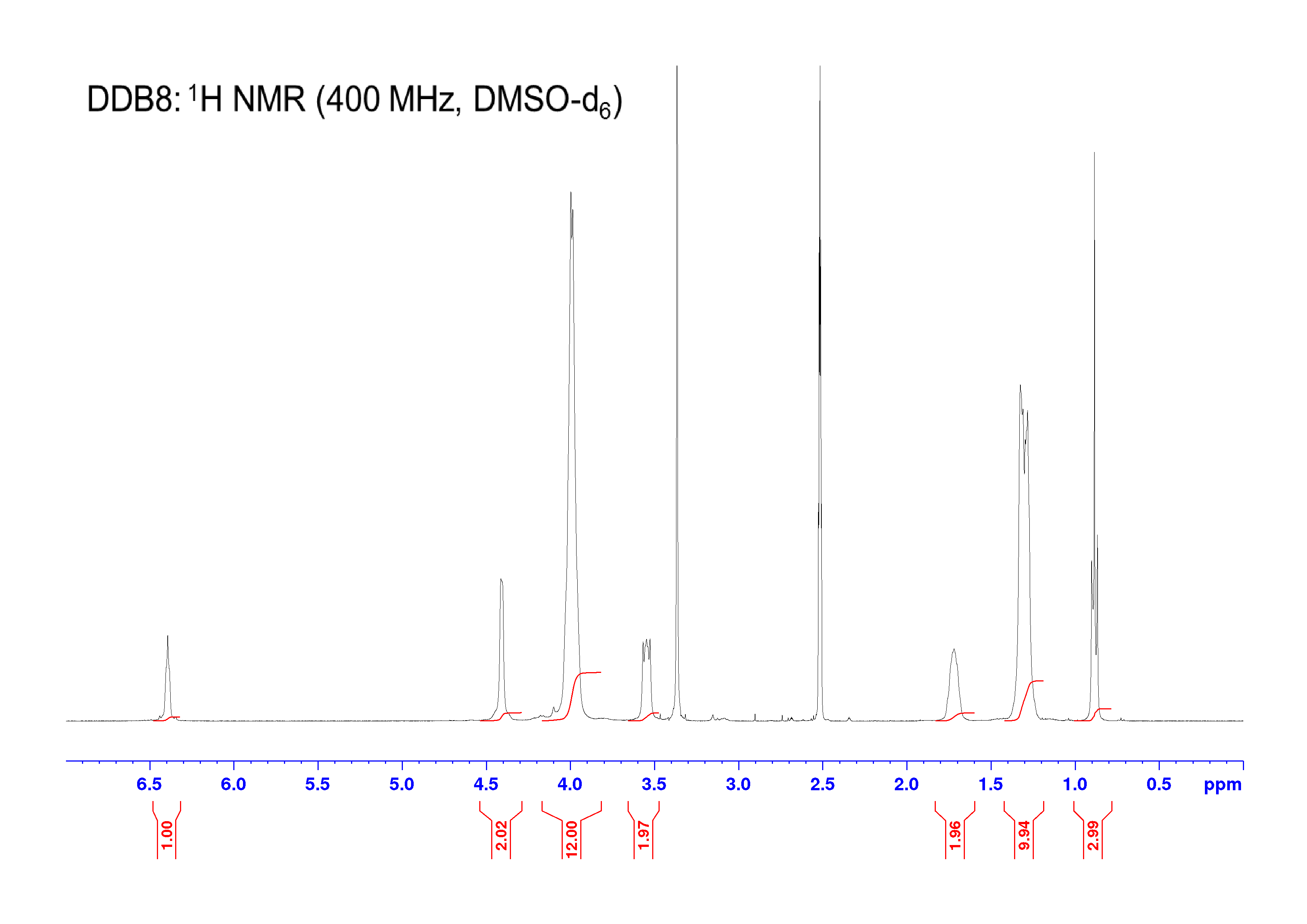
**

**DDP8: ^1^H NMR** (400 MHz, DMSO-d_6_): δ 7.77 (s, 4H), 4.98 (s, 4H), 4.00 (m, 12H), 3.91 (m, 12H), 3.48 (t, 4H), 1.66 (m, 4H), 1.28 (m, 20H), 0.88 (t, 6H).

**
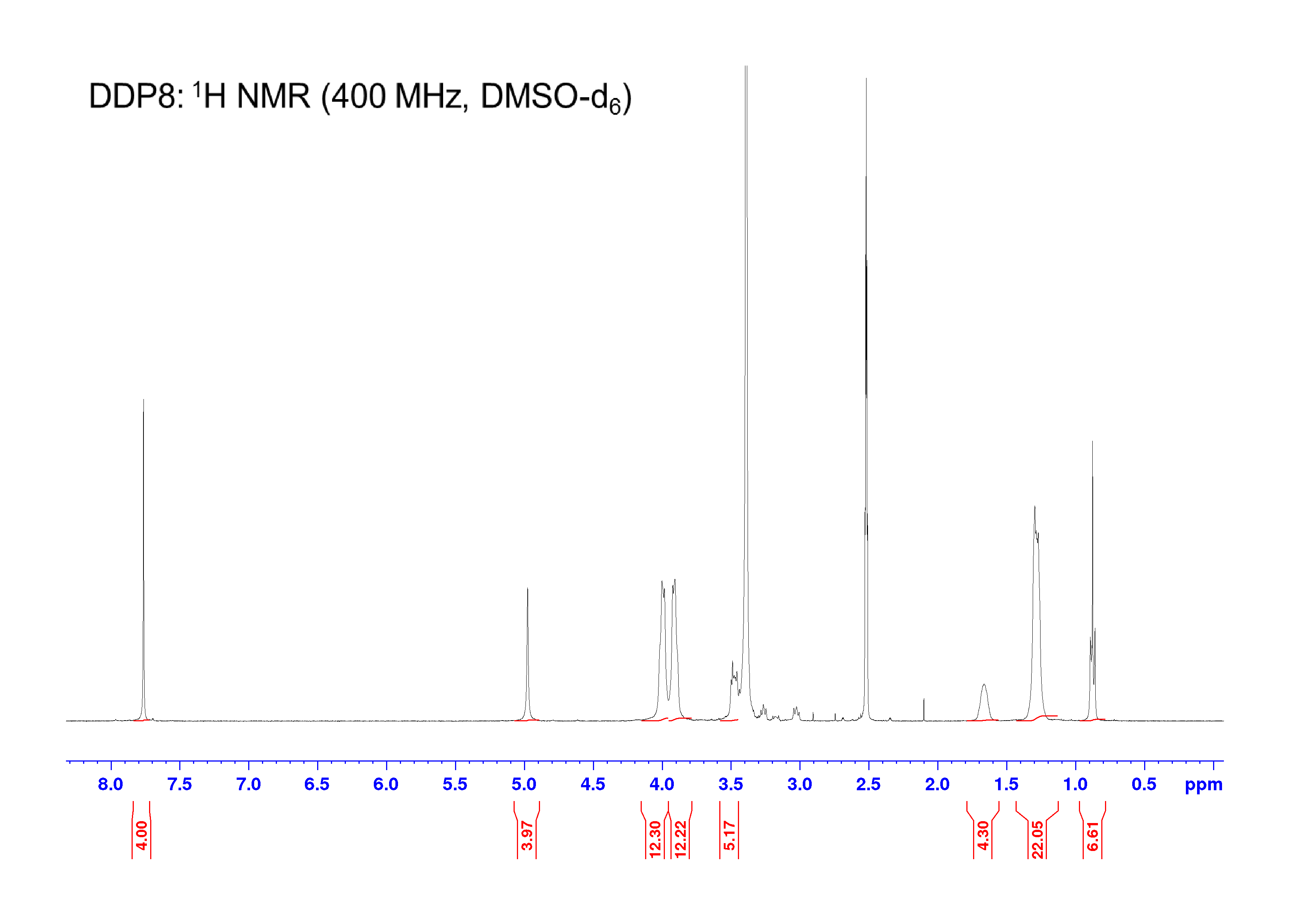
**

**DDO8: ^1^H NMR** (400 MHz, DMSO-d_6_): δ 7.84 (m, 4H), 5.28 (s, 4H), 4.10 (m, 12H), 3.85 (m, 12H), 3.51 (t, 4H), 1.65 (m, 4H), 1.28 (m, 20H), 0.88 (t, 6H).


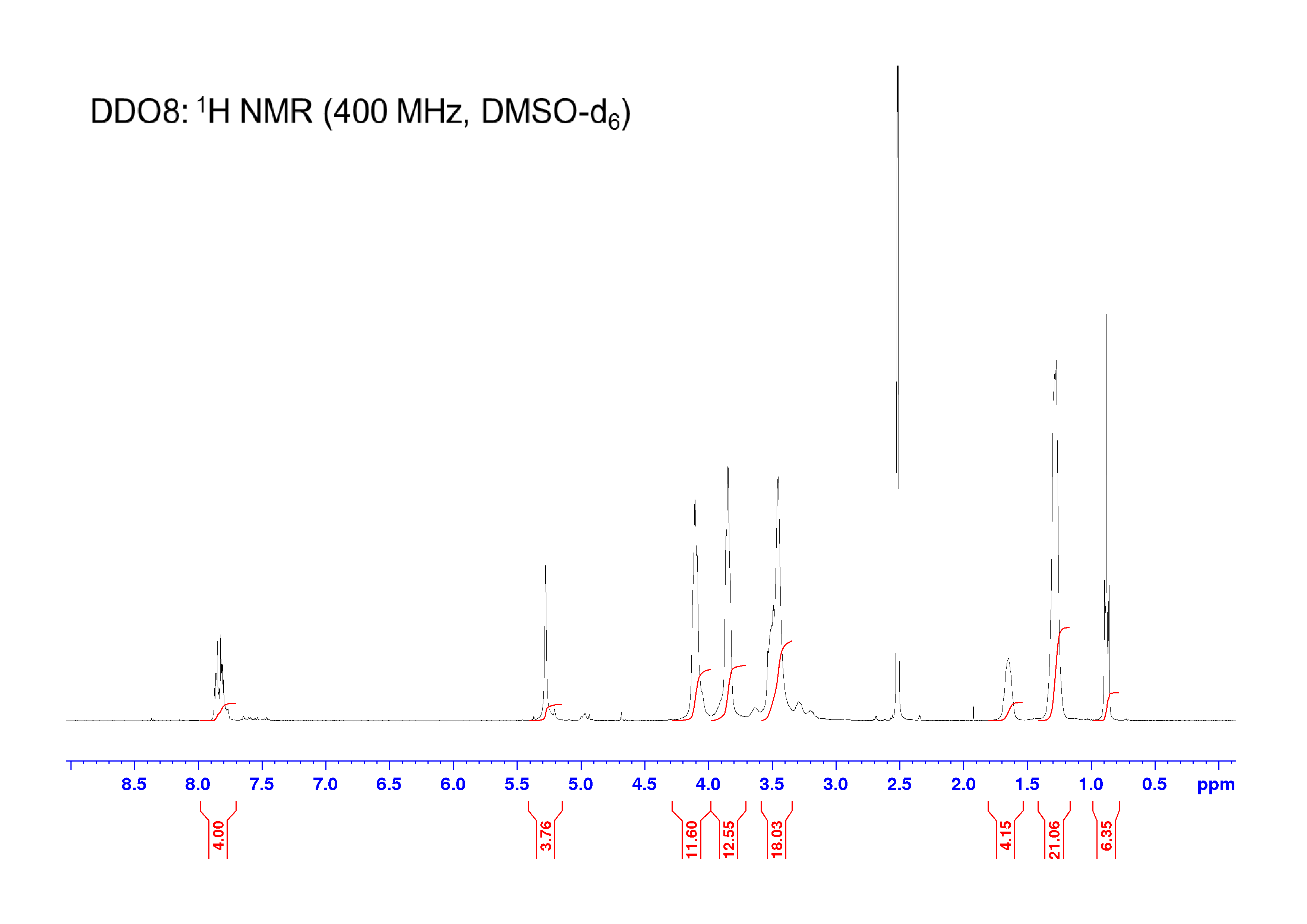


**Scheme S2.** Synthesis procedure of **IDIB8, IDIP8** and **IDIO8**.

**Synthesis of b:** A mixture of imidazole (1.36 g, 20.0 mmol) and sodium hydroxide (0.80 g, 20.0 mmol) in DMSO was heated to 90 °C for 2 hours, and then cooled to room temperature. A solution of 1-bromooctane (3.46 g, 19.0 mmol) in DMSO was added dropwise to the mixture. After stirring at room temperature for 3 hours, the mixture was heated up slowly to 65 °C for 16 hours with constant stirring. The solution obtained was mixed with water and then extracted with diethyl ether for 4 times. The diethyl ether phases were combined and dried with anhydrous sodium sulfate. Diethyl ether was removed under vacuum and **b** was obtained as a yellow liquid (2.89 g, 89%).

**b: ^1^H NMR** (400 MHz, DMSO-d_6_): δ 7.61 (s, 2H), 7.15 (s, 1H), 6.87 (s, 1H), 3.93 (t, 2H), 1.68 (m, 2H), 1.25 (m, 10H), 0.85 (t, 3H)

**Synthesis of b-1:** A solution of **b** (1.0 eq, 1.80 g, 10.0 mmol in THF) was added dropwise to a solution of trans-1,4,-dibromobut-2-ene (2.0 eq, 0.12 g in THF) at 70 °C. After overnight stirring, THF was removed from the solution under vacuum. The obtained liquid was washed with diethyl ether 3 times and then dried under vacuum (1.15 g, 29%). The synthesis of **b-2** and **b-3** was similar to **b-1.**

**b-1: ^1^H NMR** (400 MHz, DMSO-d_6_): δ 9.43 (s, 1H), 7.90 (d, 2H), 6.08 (m, 2H), 4.97 (d, 2H), 4.23 (m, 4H), 1.80 (m, 6H), 1.24 (m, 20H), 0.85 (t, 6H).

**b-2: ^1^H NMR** (400 MHz, DMSO-d_6_): δ 9.56 (s, 1H), 7.90 (d, 2H), 7.45-7.55 (m, 4H), 5.51 (s, 2H), 4.73 (s, 2H), 4.73 (s, 2H), 4.22 (t, 2H), 1.81 (m, 2H), 1.24 (m, 6H), 0.86 (t, 3H).

**b-3: ^1^H NMR** (400 MHz, DMSO-d_6_): δ 9.31 (s, 1H), 7.86 (s, 1H), 7.77 (s, 1H), 7.55 (m, 1H), 7.43 (m, 2H), 7.30 (m, 1H), 5.60 (s, 2H), 4.89 (s, 2H), 4.73 (s, 2H), 1.78 (m, 2H), 1.24 (m, 10H), 0.85 (t, 3H).

**Synthesis of IDIB8:** A mixture of **b-1** (1.15 g, 2.9 mmol) and DABCO (0.10 g, 0.9 mmol) was stirred in DMF (15 mL) at 80 °C. After overnight stirring, DMF was removed from the solution under vacuum. The obtained liquid was washed with diethyl ether 3 times and acetone 3 times, and then dried under vacuum (0.54 g, 67%). The synthesis of **IDIP8** and **IDIO8** was similar to **IDIB8.**

**IDIB8: ^1^H NMR** (400 MHz, DMSO-d_6_): δ 9.49 (s, 2H), 7.96 (t, 2H), 7.88 (t, 2H), 6.34 (m, 2H), 5.96 (m, 2H), 5.00 (d, 4H), 4.35 (d, 4H), 4.23 (t, 4H), 4.02 (s, 12H), , 1.80 (m, 4H), 1.25 (m, 20H), 0.85 (t, 6H).

**
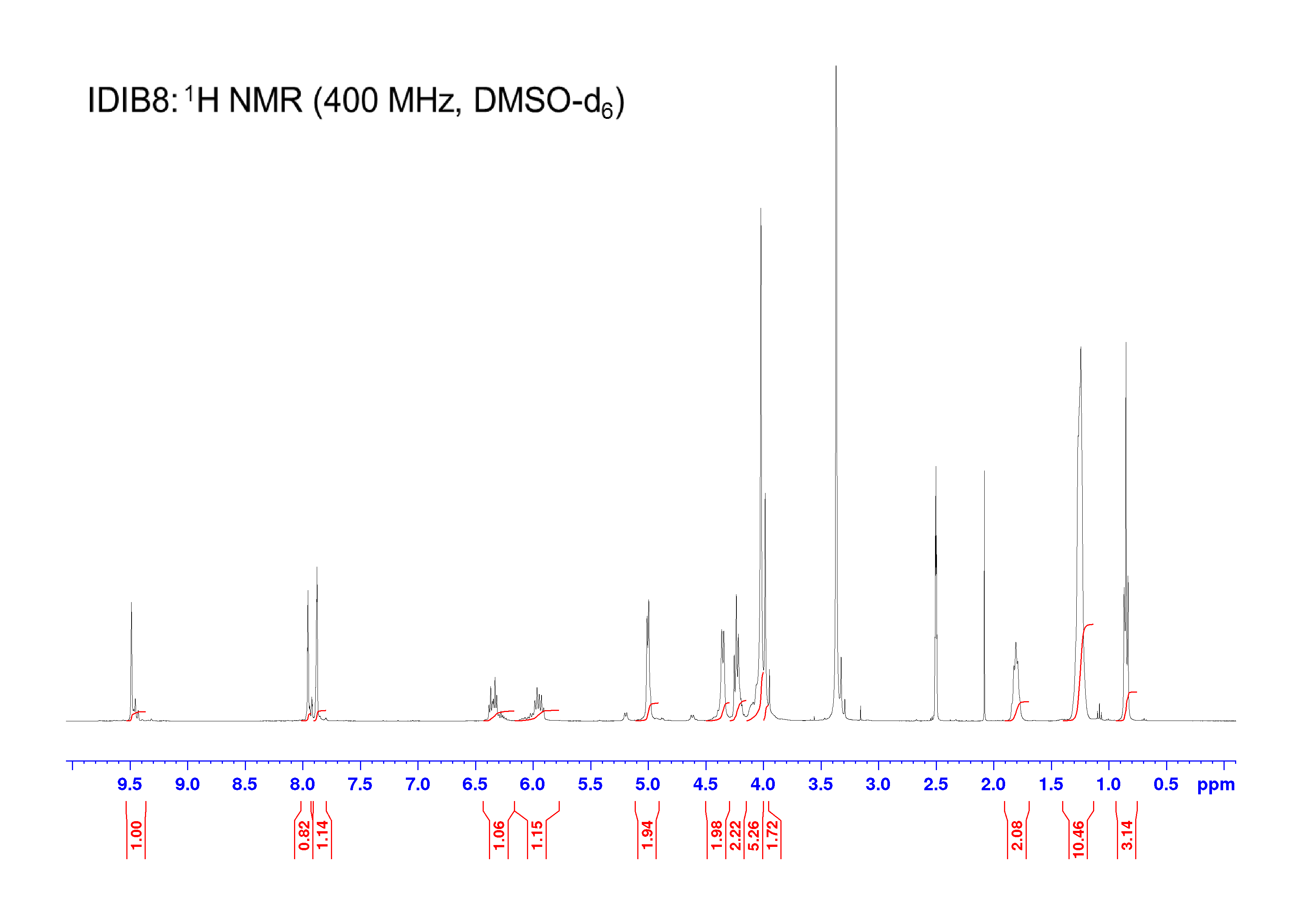
**

**IDIP8: ^1^H NMR** (400 MHz, DMSO-d_6_): δ 9.62 (s, 2H), 7.90 (t, 2H), 7.87 (t, 2H), 7.62 (s, 8H), 5.53 (s, 4H), 4.93 (s, 4H), 4.21 (t, 4H), 3.98 (m, 12H), , 1.79 (m, 4H), 1.25 (m, 20H), 0.87 (t, 6H).

**
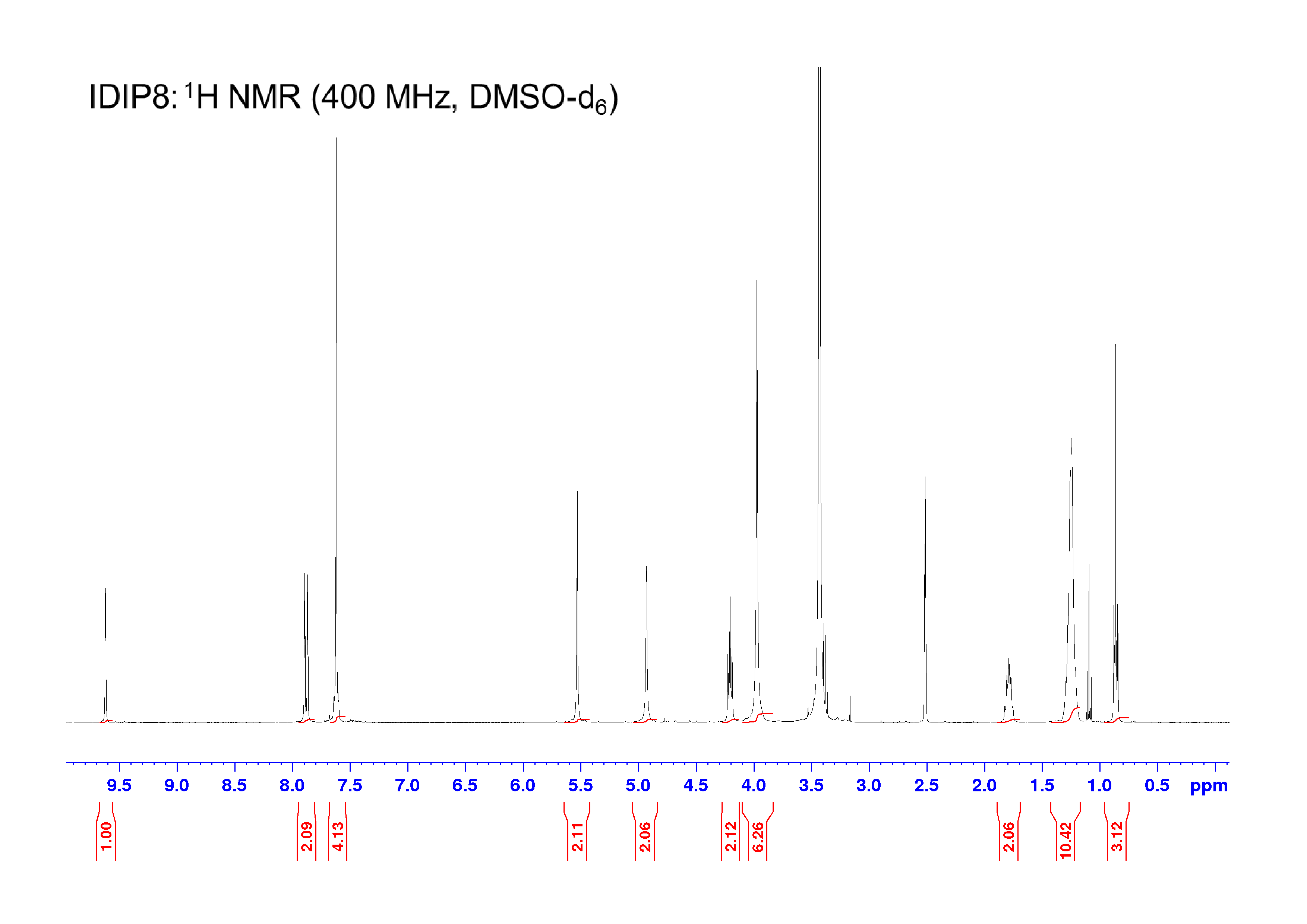
**

**IDIO8: ^1^H NMR** (400 MHz, DMSO-d_6_): δ 9.51 (s, 2H), 7.91 (t, 2H), 7.88 (t, 2H), 7.66-7.41 (m, 8H), 5.79 (s, 4H), 5.22 (s, 4H), 4.18 (t, 4H), 4.08 (m, 12H), , 1.81 (m, 4H), 1.23 (m, 20H), 0.85 (t, 6H).


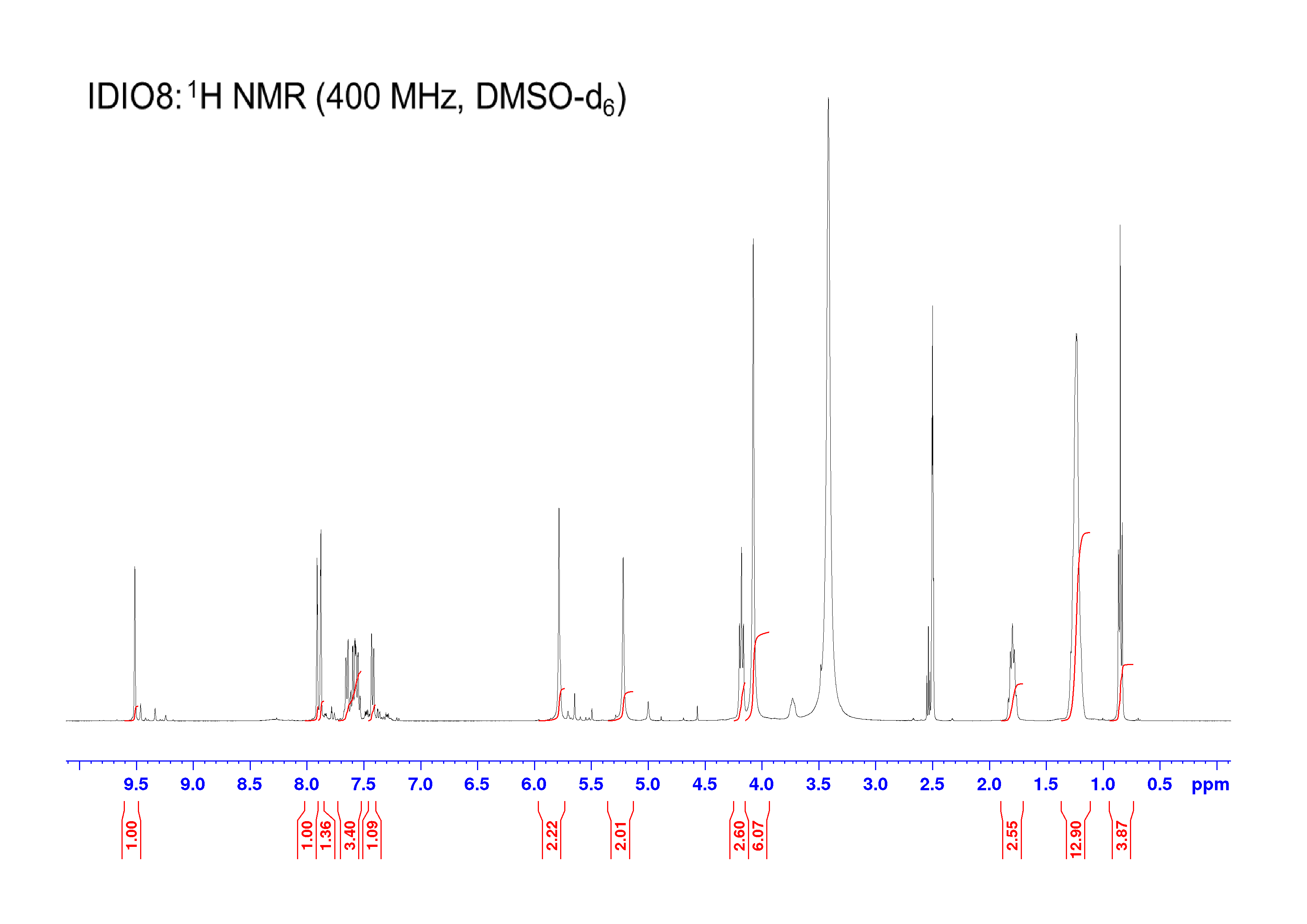


**Scheme S3.** Synthesis procedure of **IDPBX8, IDPPX8** and **IDPOX8**.

**Synthesis of 1, 2 and 3:** A solution of DABCO (8.0 eq, 5.61 g) was dissolved in MeCN (20 mL) and heated to 80 °C and a solution of α,α′-Dibromo-*p*-xylene (1.0 eq, 1.65 g) or trans-1,4,-dibromobut-2-ene (1.0 eq, 1.34 g) was added dropwise to it. The resulting solution was stirred at 90 °C for 24 hours. The solids were collected and washed with MeCN (2 x 45 mL), followed by ethyl acetate (3 x 45 mL), and then diethyl ether (2 x 45 mL) to obtain **1** (87% yield) as a white powder. The synthesis of **2** (78% yield) and **3** (86% yield) was similar to **1.**

**1:  ^1^H NMR** (400 MHz, DMSO-d_6_): δ 6.23 (t, 2H), 4.05 (d, 4H), 3.32 (t, 12H), 3.05 (t, 12H).

**2:  ^1^H NMR** (400 MHz, DMSO-d_6_): δ 7.63 (s, 4H), 4.58 (s, 4H), 3.42 (t, 12H), 3.03 (t, 12H).

**3:  ^1^H NMR** (400 MHz, DMSO-d_6_): δ 7.73 (m, 4H), 4.82 (s, 4H), 3.45 (t, 12H), 3.02 (t, 12H).

**Synthesis of IDPBX8:** **1** (0.35 g, 0.79 mmol) was dissolved in MeOH (15 mL) and heated to 60 °C. **b-2** (1.13g, 3.18 mmol) dissolved in MeOH (15 mL) was added to the solution of **1**. The mixture was stirred constantly at 40 °C for 2 days. MeOH was then removed under vacuum. The resulting white solids were washed with acetone 3 times and then dried under vacuum to obtain **IDPBX8** (50% yield). The synthesis of **IDPPX8** and **IDPOX8** was similar to **IDPBX8**.

**IDPBX8: ^1^H NMR** (400 MHz, DMSO-d_6_): δ 9.50 (s, 2H), 7.89 (m, 4H), 7.65 (m, 8H), 6.35 (t, 2H), 5.55 (s, 4H), 4.96 (s, 4H), 4.36 (s, 4H), 4.21 (t, 4H), 4.02 (s, 24H), 1.81 (m, 4H), 1.25 (m, 20H), 0.86 (t, 6H).


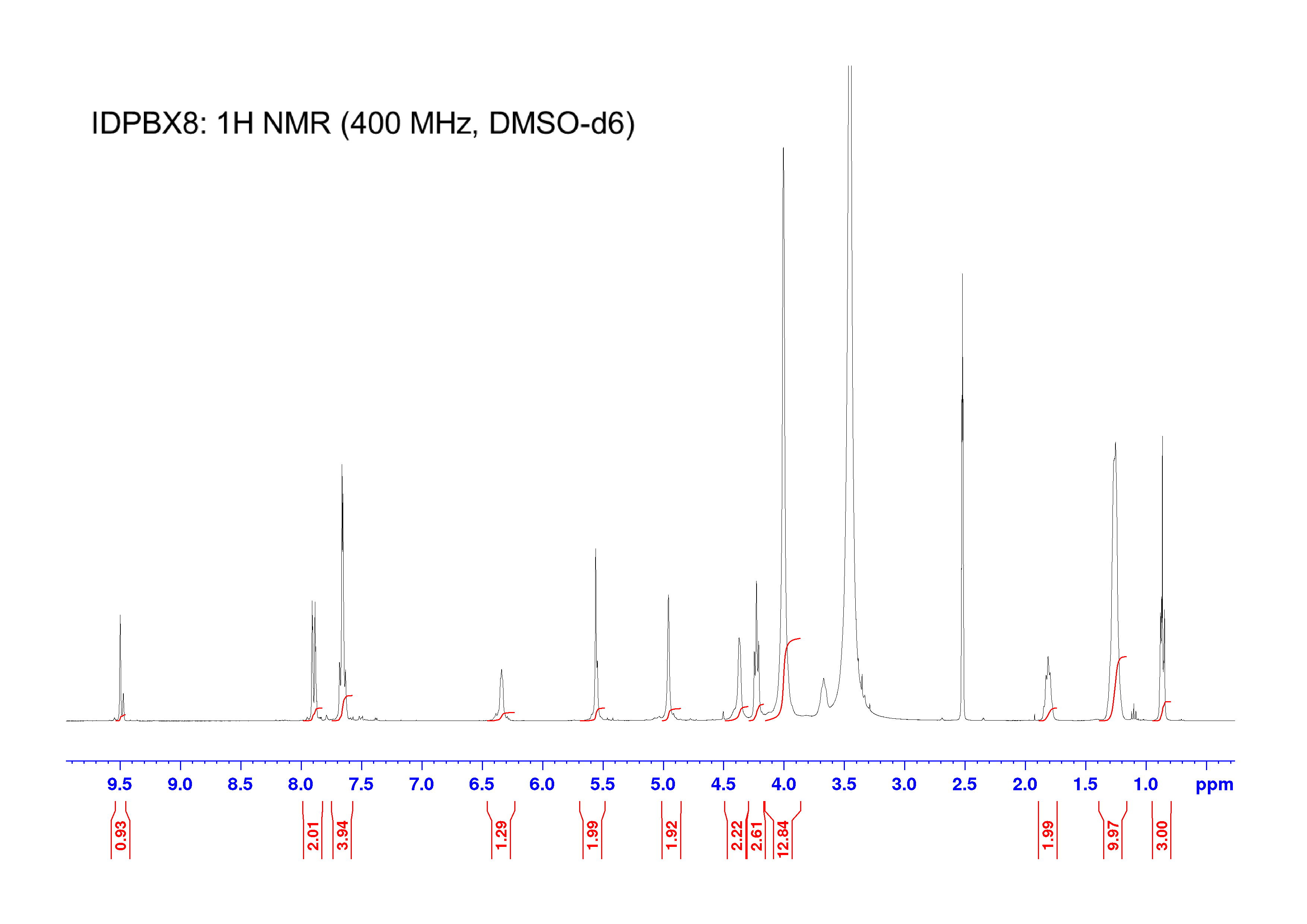


**IDPPX8: ^1^H NMR** (400 MHz, DMSO-d_6_): δ 9.48 (s, 2H), 7.87 (s, 4H), 7.75-7.66 (m, 12H), 5.53 (s, 4H), 5.05 (s, 4H), 4.98 (s, 4H), 4.21 (t, 4H), 3.97 (d, 24H), 1.82 (m, 4H), 1.25 (m, 20H), 0.87 (t, 6H).

**
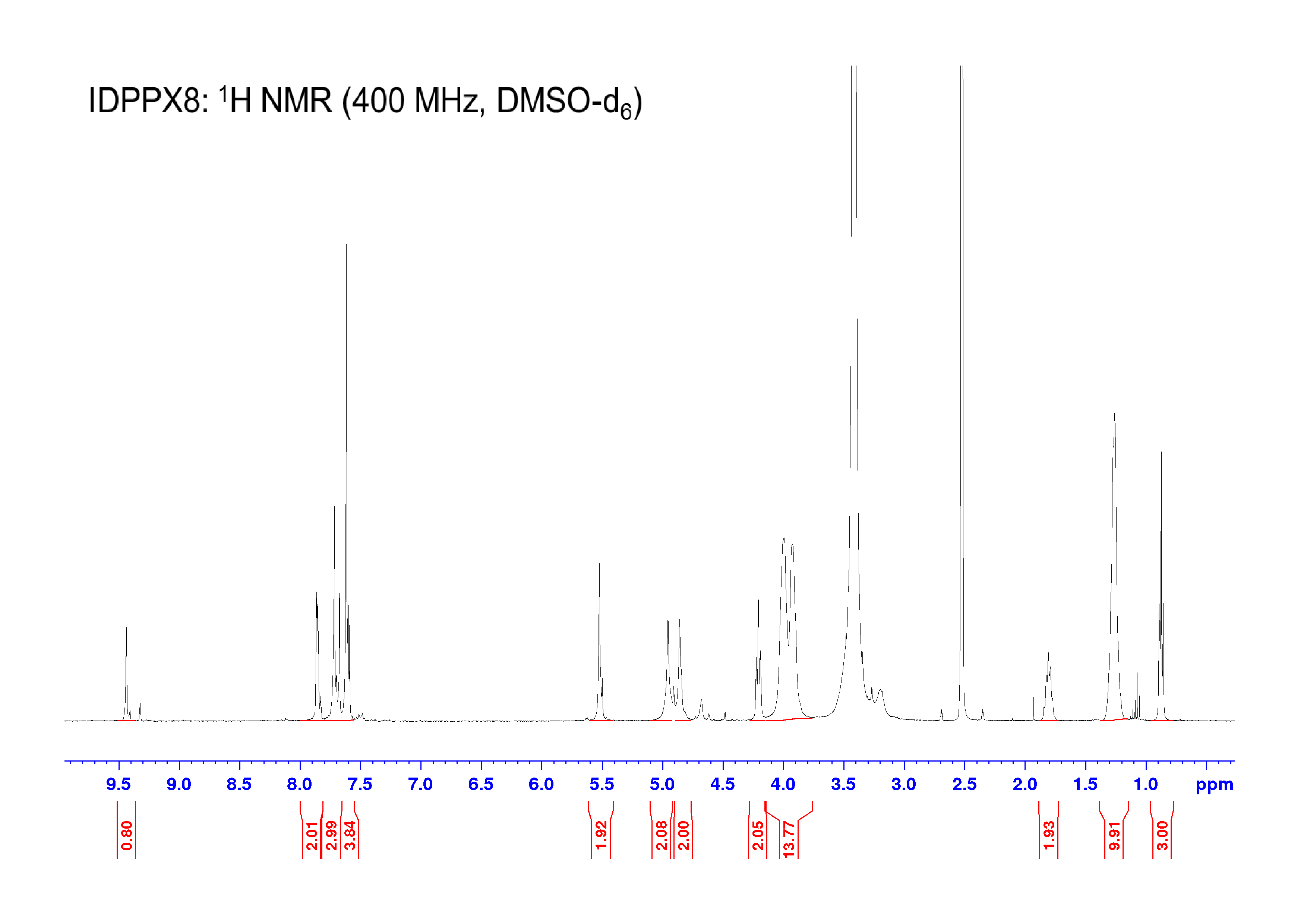
**

**IDPOX8: ^1^H NMR** (400 MHz, DMSO-d_6_): δ 9.50 (s, 2H), 7.85 (s, 4H), 7.80 (m, 4H), 7.63 (m, 8H), 5.52 (s, 4H), 5.328 (s, 4H), 4.93 (s, 4H), 4.21 (t, 4H), 4.15 (s, 12H), 3.90 (s, 12H), 1.82 (m, 4H), 1.25 (m, 20H), 0.87 (t, 6H).


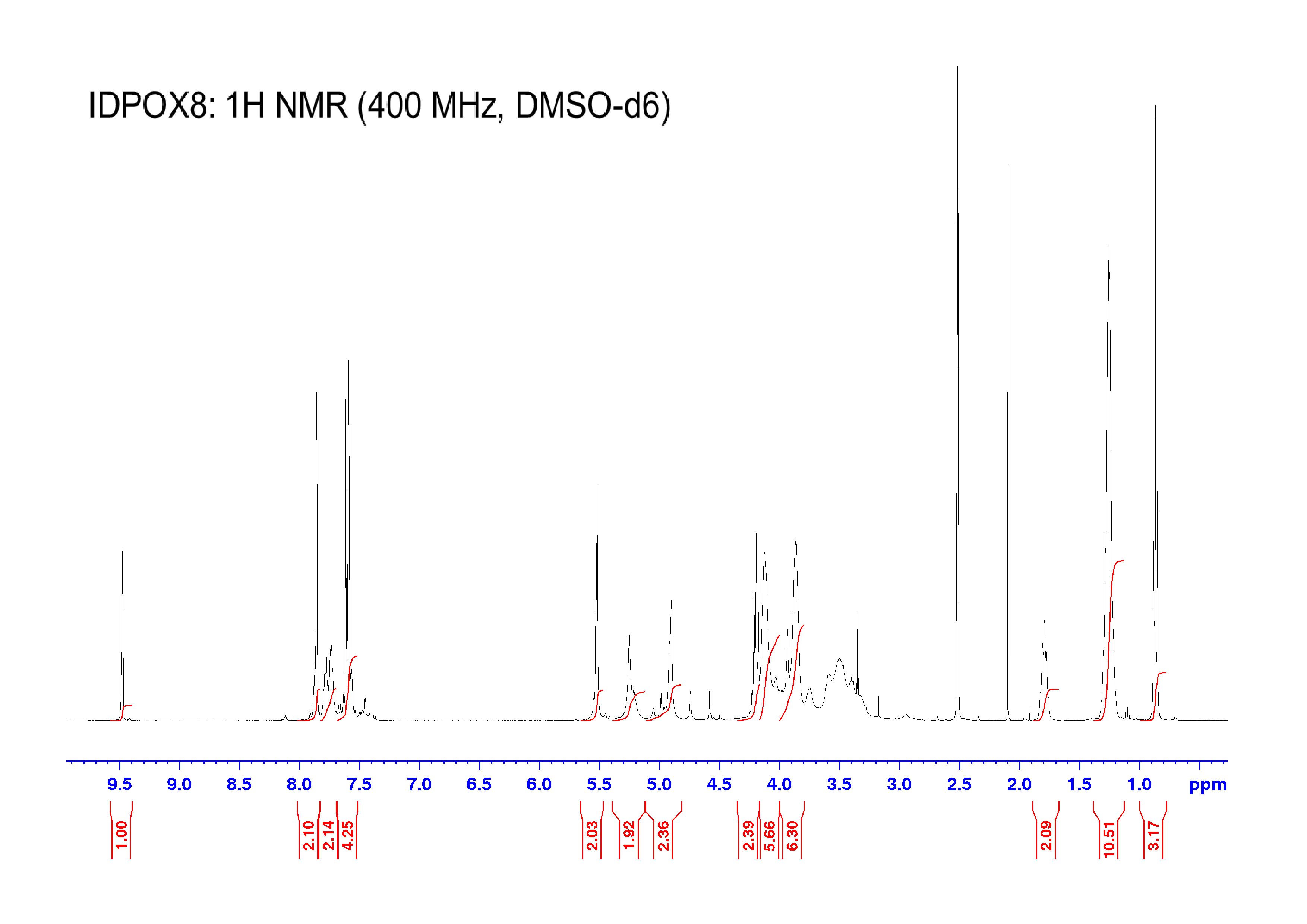


**Scheme S4.** Synthesis procedure of **IDPBXb** and **IDPPXb**.

**Synthesis of c:** The synthesis of **c** was similar to the synthesis of **b** except that **c** was obtained as colorless crystals (51% yield).

**c: ^1^H NMR** (400 MHz, DMSO-d_6_): δ 7.78 (s, 1H), 7.25-7.39 (m, 5H), 7.20 (t, 1H), 6.93 (t, 1H), 5.21 (s, 2H)

**Synthesis of c-1:** The synthesis of **c-1** was similar to the synthesis of **b-2.** A mixture of **c** (1.58 g, 10.0 mmol) and α,α′-Dibromo-*p*-xylene (13.20 g, 50.0 mmol) was stirred in THF (150 mL) at room temperature for 3 days. The mixture was concentrated by removing THF under vacuum. The obtained solid/liquid was washed with diethyl ether 3 times to remove excess α,α′-Dibromo-*p*-xylene and then extracted with acetone 4 times. The acetone solution was combined and acetone was removed under vacuum. **c-1** was obtained as light yellow liquid (41% yield).

**c-1: ^1^H NMR** (400 MHz, MeOD-d_4_): δ 9.28 (s, 1H), 7.68 (m, 2H), 7.44-7.54 (m, 9H), 5.47 (d, 4H), 4.61 (s, 2H).

**Synthesis of IDPBXb and IDPPXb:** The same procedure as the synthesis of **IDPBX8** was used to synthesize **IDPBXb** and **IDPPXb.**

**IDPBXb: ^1^H NMR** (400 MHz, DMSO-d_6_): δ 9.60 (s, 2H), 7.93 (m, 4H), 7.67 (s, 8H), 7.46 (m, 10H), 6.34 (s, 2H), 5.56 (s, 4H), 5.54 (s, 4H), 4.95 (s, 4H), 4.37 (s, 4H), 4.01 (s, 24H).

**
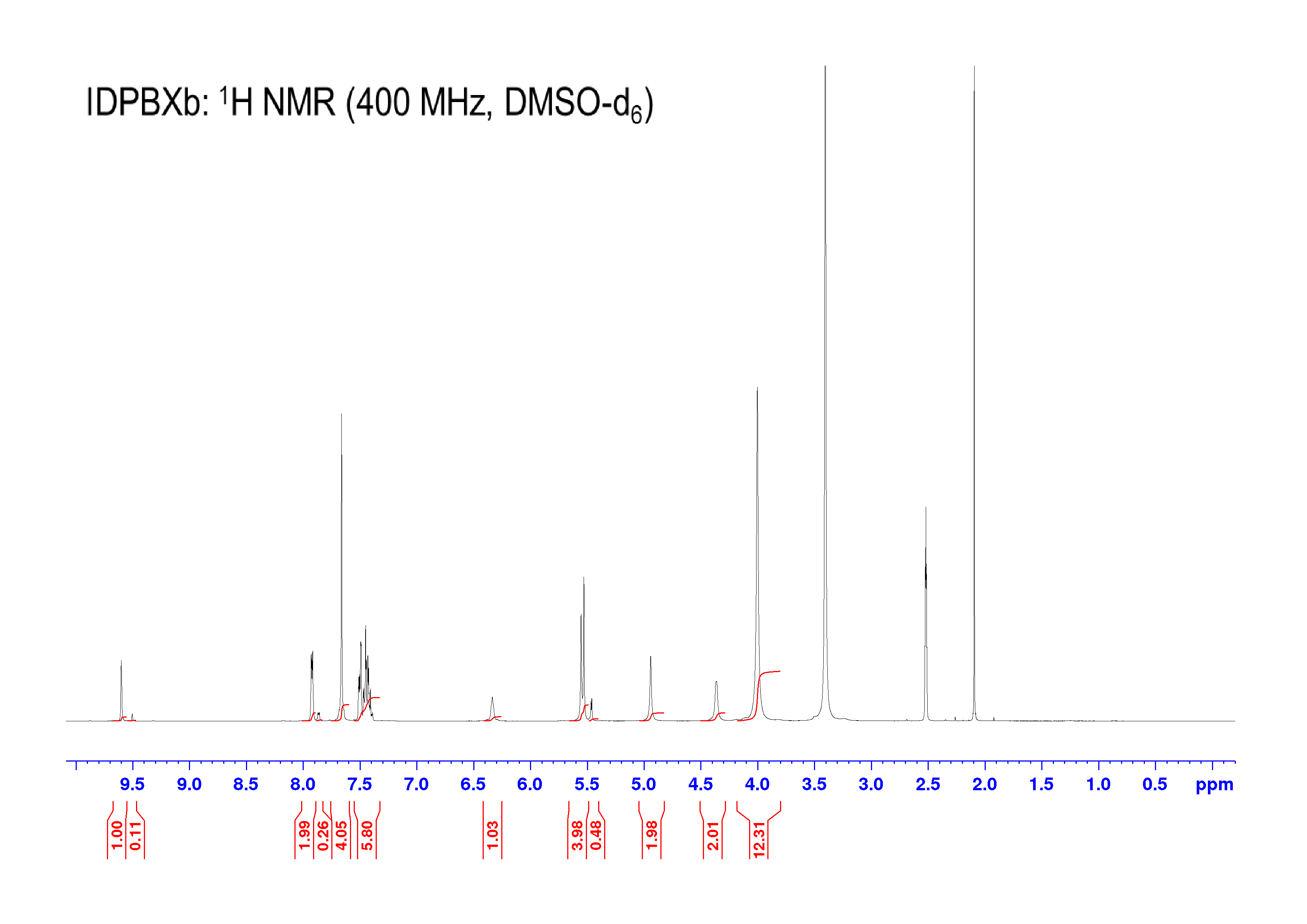
**

**IDPPXb: ^1^H NMR** (400 MHz, DMSO-d_6_): δ 9.56 (s, 2H), 7.88 (m, 4H), 7.71 (s, 4H), 7.62 (s, 8H), 7.45 (m, 10H), 5.52 (s, 4H), 5.49 (s, 4H), 4.96 (s, 4H), 4.85 (s, 4H), 3.99 (s, 12H), 3.93 (s, 12H).


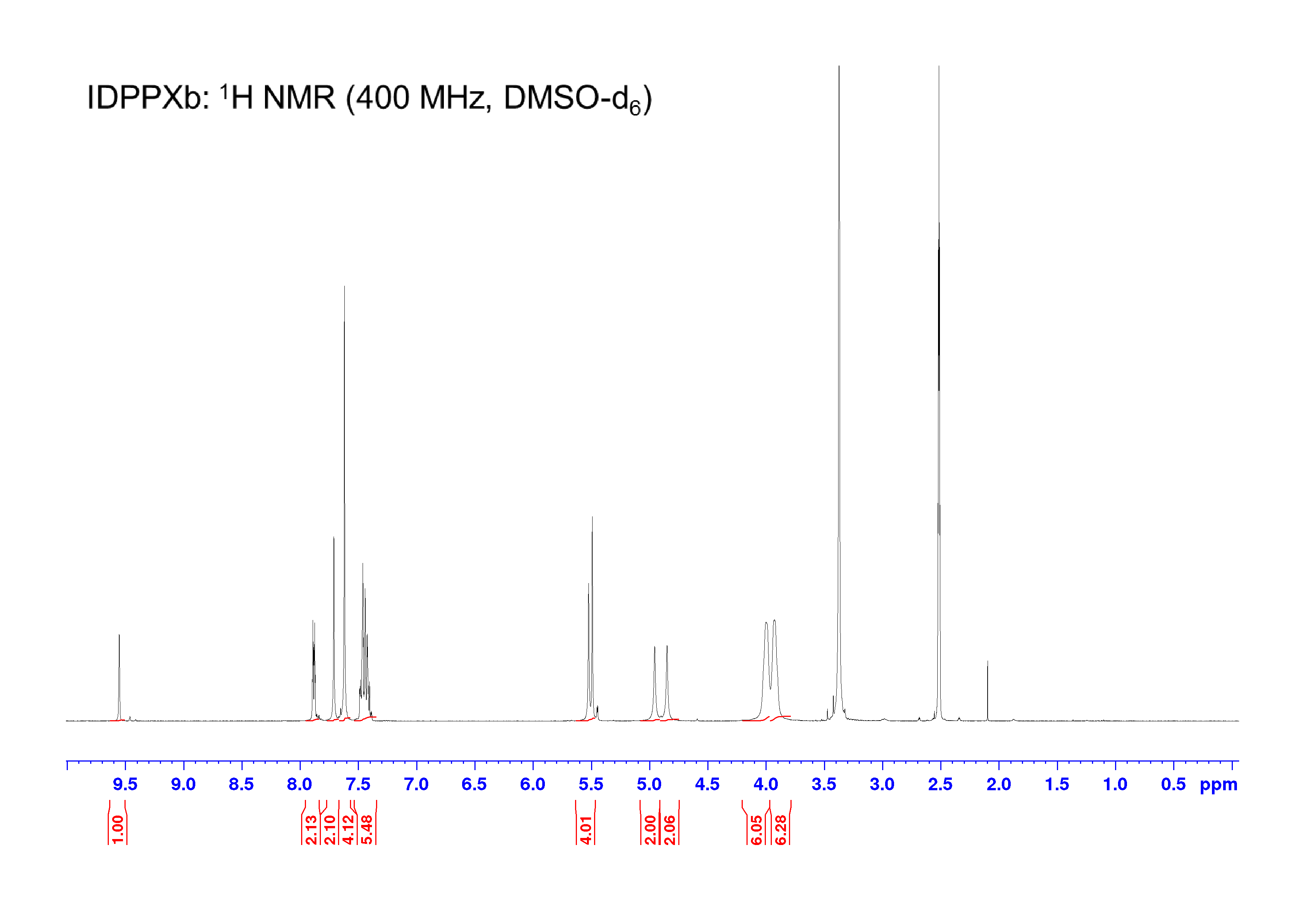


**Scheme S5.** Synthesis procedure of **IIDPBX8** and **IIDPPX8**.

**Synthesis of d-1:** The synthesis of **d** was reported previously.^1^ The purity of **d** was calculated from NMR. A solution of **d** (1.28 g, 2.96 mmol) in acetonitrile (MeCN) was added dropwise to a solution of trans-1,4-dibromo-2-butene (4.18 g, 20 mmol in MeCN).The resulting mixture was stirred at 80 °C overnight. Solvent was removed under vacuum and the resulting solids were washed with ethyl acetate. **D-1** was obtained as light yellow liquid (60% yield).

**d-1: ^1^H NMR** (400 MHz, DMSO-d_6_): δ 9.42 (s, 1H), 9.36 (s, 1H), 7.86 (t, 1H), 7.84 (d, 2H), 7.76 (t, 1H), 7.50 (m, 4H), 6.05 (m, 2H), 5.47 (d, 4H), 4.91 (d, 2H), 4.18 (m, 4H), 1.78(m, 2H),1.24 (m, 10H), 0.86 (t, 3H).

**Synthesis of IIDPBX8 and IIDPPX8:** A solution of **d-1** (0.748 g, 1.16 mmol) in DMF was mixed with a solution of DABCO (0.04 g, 0.38 mmol). The resulting mixture was stirred at 90 °C for 16 h. After reaction, the solvent was removed under vacuum. And the solids obtained were dissolved in methanol and re-precipitated with acetone twice. After washing with acetone twice, the product was dried under vacuum. **IIDPBX8** was obtained as white powder (28% yield).

The synthesis of **d-2** was reported previously^2^. **IIDPPX8** was synthesized under similar condition as **IIDPBX8.**

**IIDPBX8: ^1^H NMR** (400 MHz, DMSO-d_6_): δ 9.64 (s, 2H), 9.44 (s, 2H), 7.99 (t, 8H), 7.53 (m, 8H), 6.37 (m, 2H), 6.02 (m, 2H), 5.52 (d, 8H), 5.02(d, 4H), 4.37 (d, 4H), 4.20 (t, 4H), 4.03 (s, 12H), 1.80 (m, 4H), 1.25 (m, 20H), 0.86 (t, 6H).

**
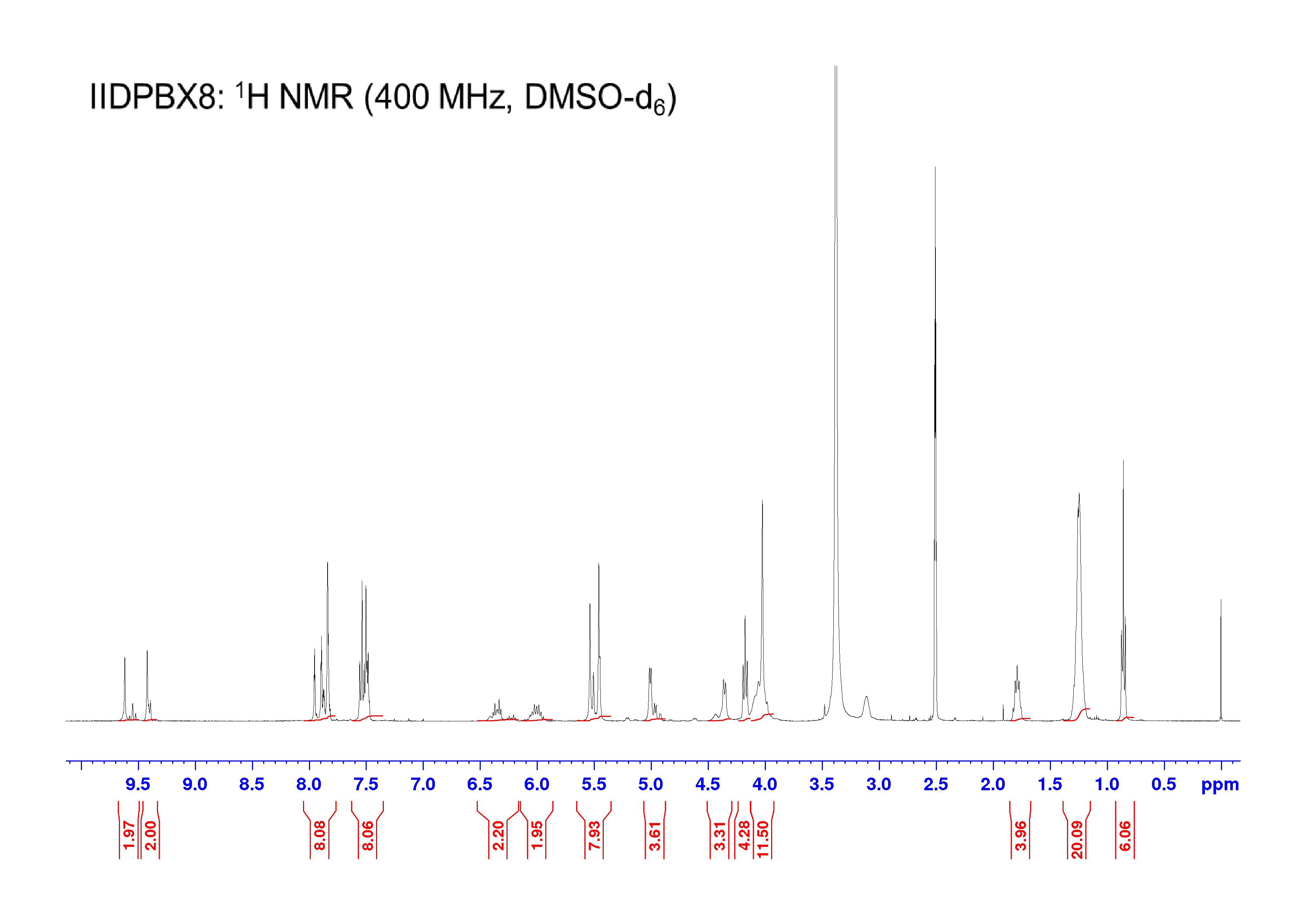
**

**IIDPPX8: ^1^H NMR** (400 MHz, DMSO-d_6_): δ 9.79 (s, 2H), 9.54 (s, 2H), 7.88 (t, 8H), 7.64 (s, 8H), 6.53 (q, 8H), 5.52 (t, 12H), 4.93(s, 4H), 4.19 (t, 4H), 3.99 (s, 12H), 1.80 (m, 4H), 1.25 (m, 20H), 0.86 (t, 6H).

**
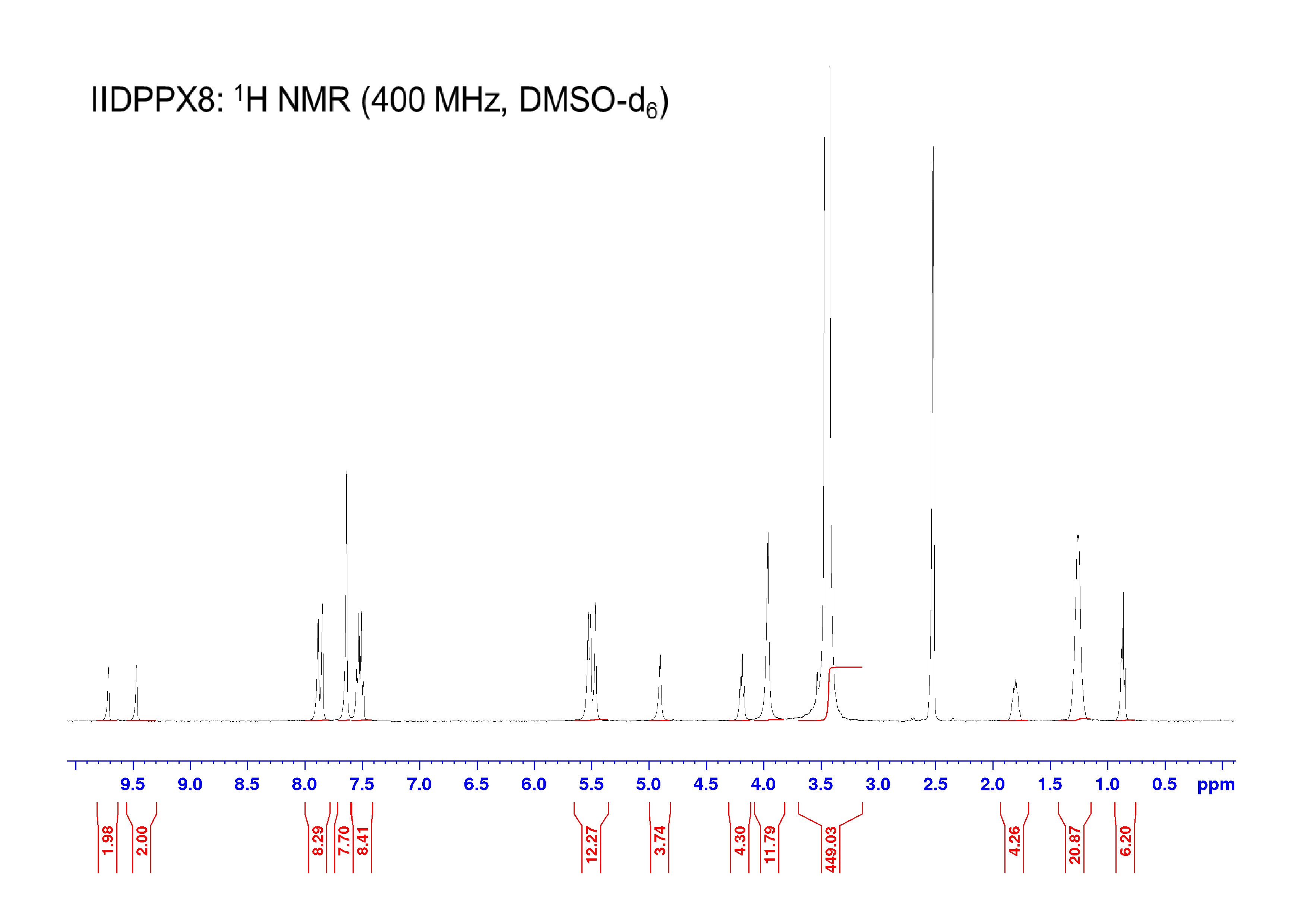
**

**Scheme S6.** Synthesis procedure of **IIDPPBX8**.

**Synthesis of IIDPPBX8:** A solution of **1** (0.096 g, 0.22 mmol) in MeOH was mixed with a solution of **d-2** (0.77 g, 1.10 mmol). The mixture was stirred constantly at 60 °C for 48 hours. MeOH was then removed under vacuum and the resulting solids were washed with acetone twice and DMF twice. After being re-precipitated with MeOH and diethyl ether twice, the resulting solids were dried under vacuum. **IIDPPBX8** was obtained as white solids (58%).

**IIDPPBX8**: **^1^H NMR** (400 MHz, DMSO-d_6_): δ 9.65 (s, 2H), 9.46 (s, 2H), 7.92 (m, 4H), 7.86 (d, 4H), 7.67 (s, 8H), 7.53 (m, 8H), 6.34 (s, 2H), 5.55 (t, 12H), 4.97 (s, 4H), 4.37 (s, 4H), 4.19 (t, 4H), 4.01 (s, 24 H), 1.80 (m, 4H), 1.26 (d, 20 H), 0.86 (t, 6H).


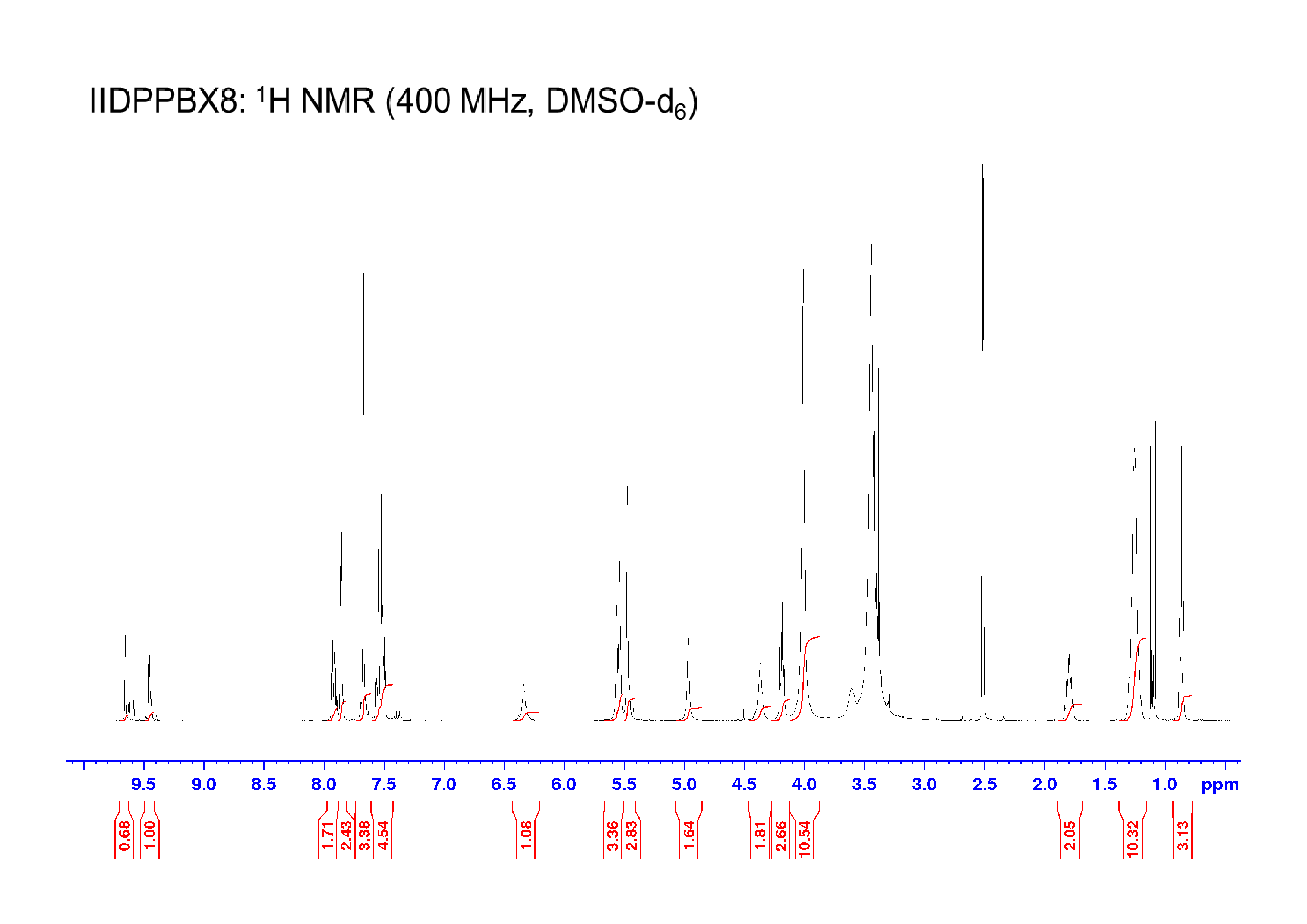


**Table S1.** Antimicrobial activity (MIC, µg mL^-1^), the fractional inhibitory concentration index (FIC) with Fluconazole, haemolytic property (HC_10_, µg mL^-1^), and critical micelle concentration (CMC, µg mL^-1^) of the ammonium-imidazolium oligomers.

| **Sample name** | **MIC (µg mL^-1^) ^a)^** | | | | **HC_10_**  **(µg mL^-1^)** | **FIC** | **CMC**  **(µg mL^-1^)** |
| --- | --- | --- | --- | --- | --- | --- | --- |
|  | ***S.A.*** | ***E.C.*** | ***P.A.*** | ***C.A.*** |  |  |  |
| **DDB8** | 4 | 8 | 125 | 125(16) | >2000 | 0.25 | 1552 |
| **DDP8** | 16 | 16 | 2000 | 500(31) | >2000 | 0.19 | 1896 |
| **DDO8** | 4 | 8 | 62 | 125(16) | >2000 | 0.38 | 3038 |
| **IDIP8** | 2 | 16 | 1000 | 125(8) | >2000 | 0.25 | 985 |
| **IDIB8** | 8 | 16 | 500 | >500(31) | >2000 | 0.25 | 1829 |
| **IDIO8** | 8 | 16 | 250 | 62(31) | >2000 | 0.25 | 1667 |
| **IDPBX8** | 16 | 31 | 62 | 8-31(2-8) | >2000 | 0.38 | 1456 |
| **IDPPX8** | 8 | 62 | 500 | 62(8-31) | >2000 | 0.25 | 1552 |
| **IDPOX8** | 4 | 8 | 62 | 125(16) | >2000 | 0.25 | 1367 |
| **IDPBXb** | 125 | 62 | >1000 | >1000 | >2000 | na | 1732 |
| **IDPPXb** | 125 | 62 | >1000 | (1000) | na | 0.5 | 1860 |
| **ITPPX8** | 4 | 16 | 500 | 2000 | >2000 | 0.25 | 1342 |
| **IIDPBX8** | 4 | 62 | 250 | (500) | >2000 | 0.31 | 291 |
| **IIDPPX8** | 4 | 16 | 125 | 125(16) | >2000 | 0.38 | 451 |
| **IIDPPBX8** | 8 | 31 | 125 | 62(8) | >2000 | 1.00 | 727 |
| **IBN-C8** ^17^ | 4 | 8 | 16 | 125(16) | >2000 | 2.00 | 226 |
| Fluconazole | - | - | - | 31(2-8) | - | **-** | **-** |
| Itraconazole | - | - | - | 16(2-4) | - | **-** | **-** |
| Voriconazole | - | - | - | >125(2-4) | - | **-** | **-** |

*EC* (*E. coli*), *SA* (*S. aureus*), *P A* (*P. aeruginosa*), *CA* (*C. albicans*), Flu (fluconazole), Itra (itraconazole), Vori (voriconazole).

^a^ MIC tested against ~3×10^8^ CFU mL^-1^ of bacteria or 10^6^ CFU mL^-1^ of fungi. The MIC endpoints were defined as the lowest concentration that causes at least 90% decrease in optical density. For MIC tested against *C. albicans*, the lowest concentration that inhibited at least 50% fungal growth was listed in brackets.

^b^ The FIC of a compound with fluconazole was calculated using the lowest concentration of a compound that inhibited at least 50% fungal growth since fluconazole is fungal-static.

**Computational method**

All MD simulations were performed using the GROMACS 4.5.3 suite of programs^3^. The amber03 force field^4^ was used to describe the drug and oligomer molecules. Water molecules were described using the TIP5P water model^5^. Periodic boundary conditions were implemented in all systems. A cutoff of 1 nm was implemented for the Lennard–Jones and the direct space part of the Ewald sum for Coulombic interactions. The Fourier space part of the Ewald splitting was computed by using the particle-mesh-Ewald (PME) method^6^, with a grid length of 0.16 nm on the side and a cubic spline interpolation. Each sample was run for 20ns. The initial coordinates of the drug and oligomer molecules were made from Gaussview^7^ and optimized by density functional theory using M06-2X^8^ functional and 6-31g(d) basis set^9^ as built in Gaussian 09 package ^10^.


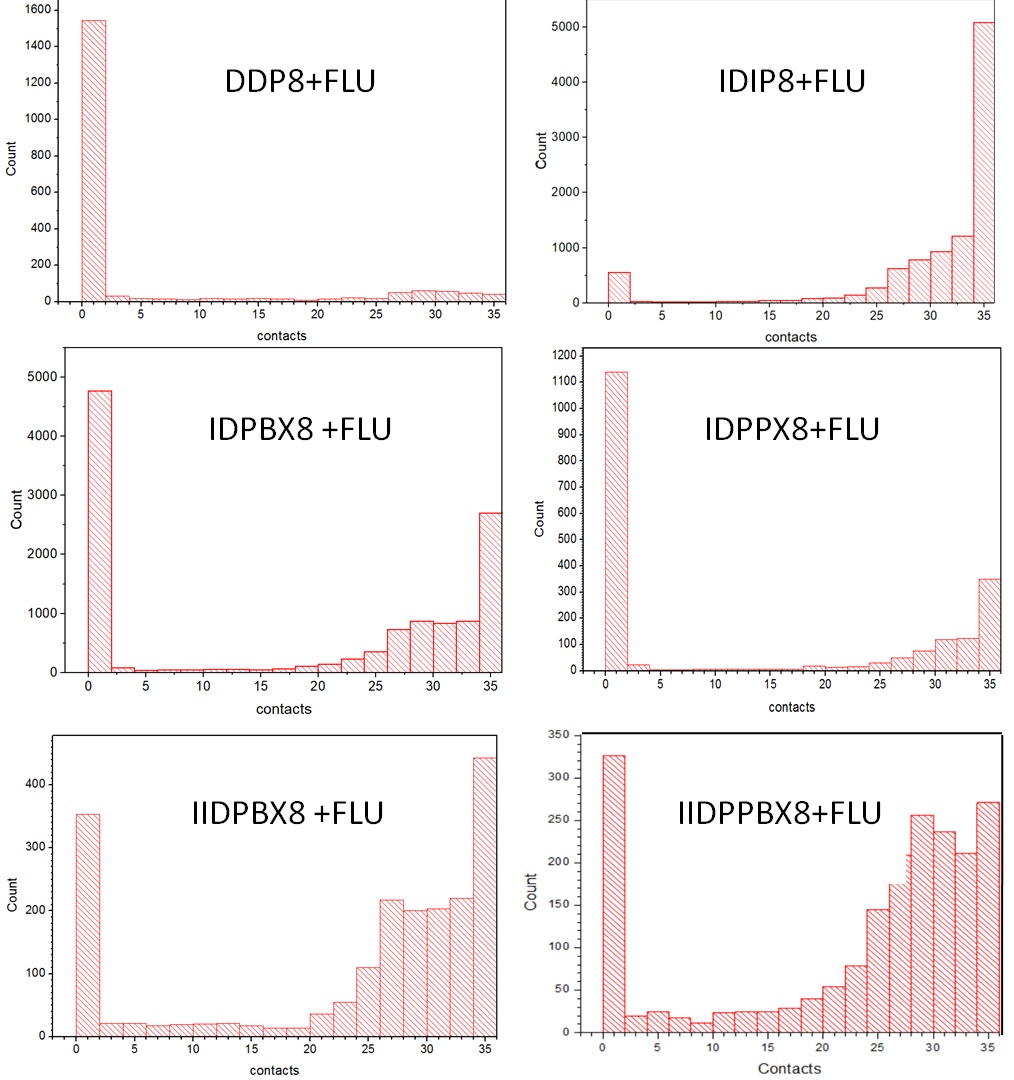


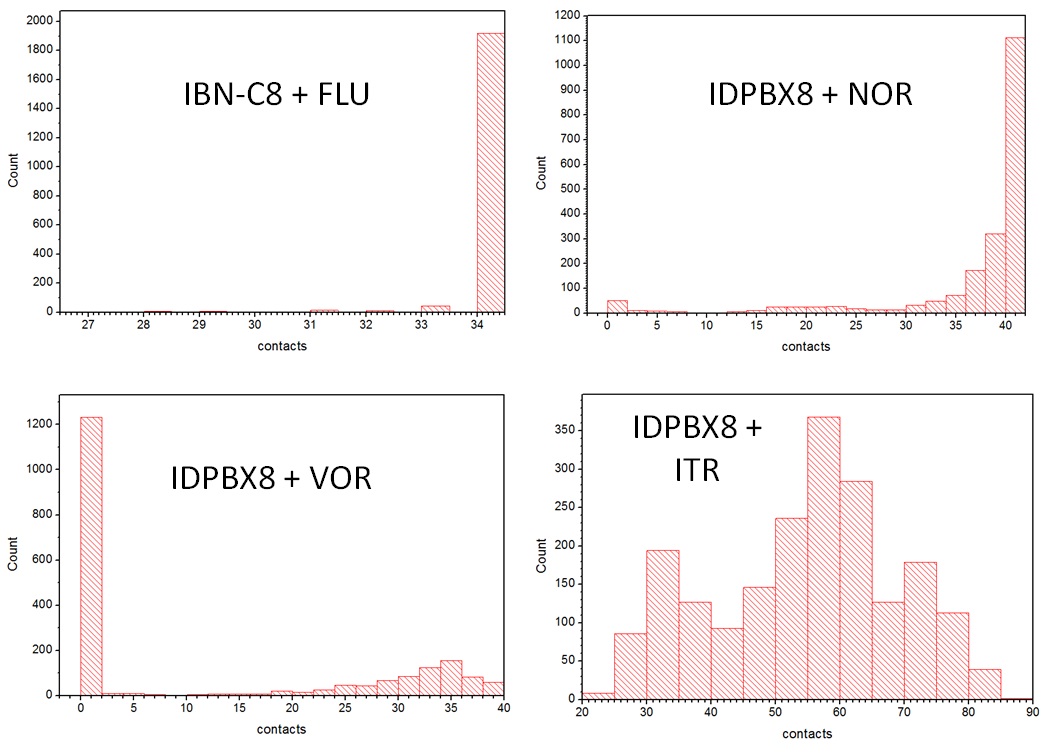


**Figure S1**. The number of contacts of atoms between oligomer and drug molecules within 6 Å. Oligomer and drug molecules are separated when the peak is at contacts=0, oligomer and drug molecules are strongly attached when the peak is at maximum contacts.


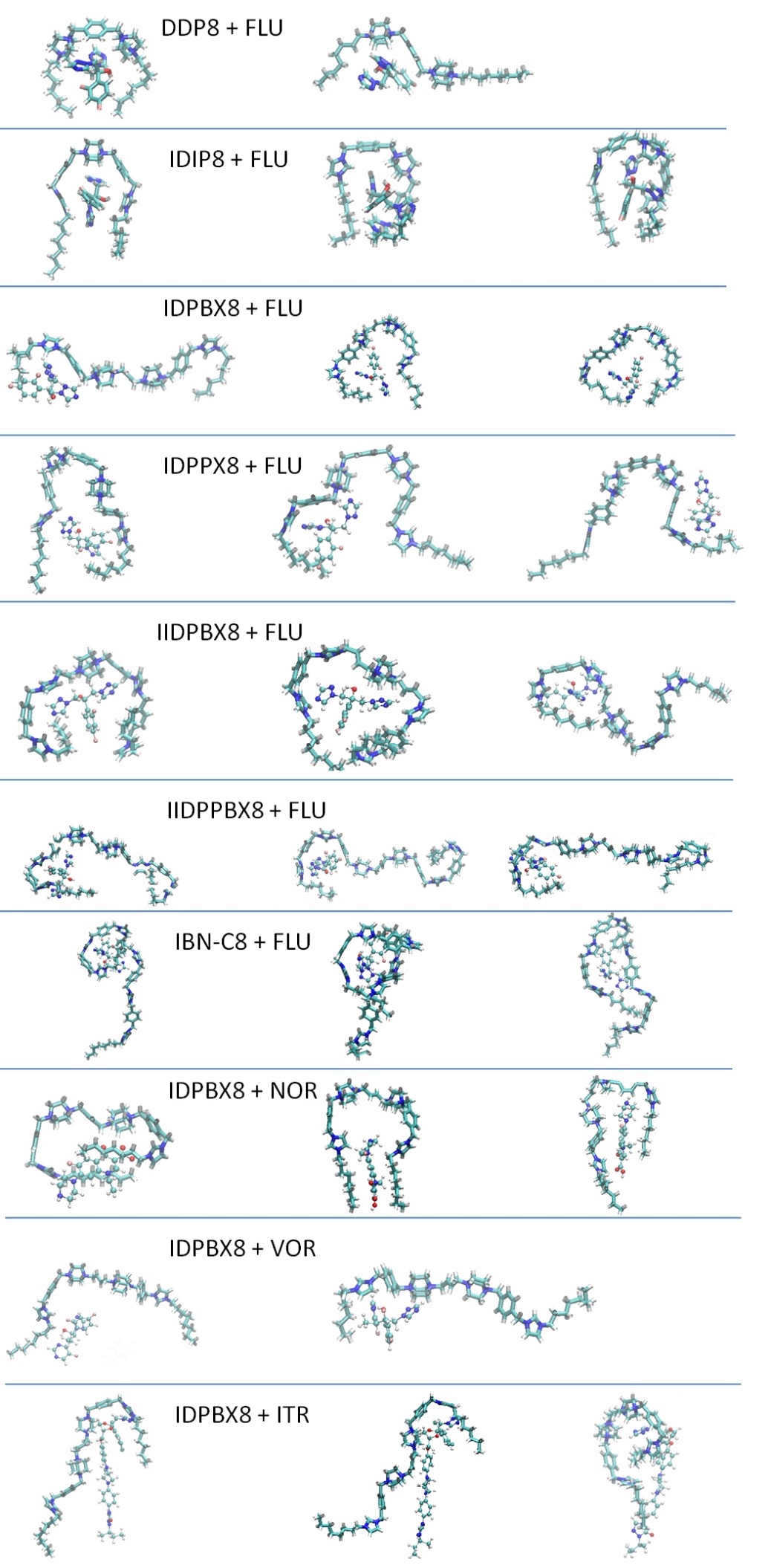


**Figure S2**. The representative structures for oligomer-drug binding states during 20ns MD runs.

**References**

1. S. N. Riduan, Y. Yuan, F. Zhou, J. Leong, H. Su, Y. Zhang, Ultrafast killing and self‐gelling antimicrobial imidazolium oligomers. *Small* 2016, **12**, 1928-1934.
2. Y. Yuan, Y. Zhang, Synthesis of imidazolium oligomers with planar and stereo cores and their antimicrobial applications. *ChemMedChem* 2017, **12**, 835-840.
3. D. Van der Spoel, E. Lindahl, B. Hess, G. Groenhof, A. E. Mark, H. J. C. Berendsen, GROMACS: Fast, flexible, and free. *Journal of Computational Chemistry* 2005, **26**, 1701-1718.
4. Y. Duan, C. Wu, S. Chowdhury, M. C. Lee, G. M. Xiong, W. Zhang, R. Yang, P. Cieplak, R. Luo, T. Lee, J. Caldwell, J. M. Wang, P. Kollman, A point-charge force field for molecular mechanics simulations of proteins based on condensed-phase quantum mechanical calculations. *Journal of Computational Chemistry* 2003, **24**, 1999-2012.
5. W. L. Jorgensen, J. Chandrasekhar, J. D. Madura, R. W. Impey, M. L. Klein, COMPARISON OF SIMPLE POTENTIAL FUNCTIONS FOR SIMULATING LIQUID WATER. *Journal of Chemical Physics* 1983, **79**, 926-935.
6. T. Darden, D. York, L. Pedersen, *J. Chem. Phys.* 1993, **98**, 10089–10092.
7. GaussView, Version 5, R. Dennington, T. Keith, and J. Millam, Semichem Inc., Shawnee Mission, KS, 2009.
8. Y. Zhao, D. G. Truhlar, The M06 suite of density functionals for main group thermochemistry, thermochemical kinetics, noncovalent interactions, excited states, and transition elements: two new functionals and systematic testing of four M06-class functionals and 12 other functionals. *Theoretical Chemistry Accounts* 2008, **120**, 215-241.
9. R. Ditchfield, W. J. Hehre, J. A. Pople, *Journal of Chemical Physics* 1971, **54**, 724.
10. Gaussian 09, Revision A.02, M. J. Frisch, G. W. Trucks, H. B. Schlegel, G. E. Scuseria, M. A. Robb, J. R. Cheeseman, G. Scalmani, V. Barone, G. A. Petersson, H. Nakatsuji, X. Li, M. Caricato, A. Marenich, J. Bloino, B. G. Janesko, R. Gomperts, B. Mennucci, H. P. Hratchian, J. V. Ortiz, A. F. Izmaylov, J. L. Sonnenberg, D. Williams-Young, F. Ding, F. Lipparini, F. Egidi, J. Goings, B. Peng, A. Petrone, T. Henderson, D. Ranasinghe, V. G. Zakrzewski, J. Gao, N. Rega, G. Zheng, W. Liang, M. Hada, M. Ehara, K. Toyota, R. Fukuda, J. Hasegawa, M. Ishida, T. Nakajima, Y. Honda, O. Kitao, H. Nakai, T. Vreven, K. Throssell, J. A. Montgomery, Jr., J. E. Peralta, F. Ogliaro, M. Bearpark, J. J. Heyd, E. Brothers, K. N. Kudin, V. N. Staroverov, T. Keith, R. Kobayashi, J. Normand, K. Raghavachari, A. Rendell, J. C. Burant, S. S. Iyengar, J. Tomasi, M. Cossi, J. M. Millam, M. Klene, C. Adamo, R. Cammi, J. W. Ochterski, R. L. Martin, K. Morokuma, O. Farkas, J. B. Foresman, and D. J. Fox, Gaussian, Inc., Wallingford CT, 2016.
